# Linking Intertidal and Subtidal Food Webs: Consumer-Mediated Transport of Intertidal Benthic Microalgal Carbon

**DOI:** 10.1371/journal.pone.0139802

**Published:** 2015-10-08

**Authors:** Chang-Keun Kang, Hyun Je Park, Eun Jung Choy, Kwang-Sik Choi, Kangseok Hwang, Jong-Bin Kim

**Affiliations:** 1 School of Environmental Science & Engineering, Gwangju Institute of Science and Technology, Gwangju, Republic of Korea; 2 Department of Marine Bioscience, Gangneung-Wonju Nattional University, Gangneung, Republic of Korea; 3 Korea Polar Research Institute, Korea Institute of Ocean Science and Technology, Incheon, Republic of Korea; 4 Faculty of Marine Biomedical Science, Jeju National University, Jeju, Republic of Korea; 5 Fisheries Resources Management Division, National Fisheries Research & Development Institute (NFRDI), Busan, Republic of Korea; 6 Subtropical Fisheries Research Center, NFRDI, Jeju, Republic of Korea; Texas A&M University at Galveston, UNITED STATES

## Abstract

We examined stable carbon and nitrogen isotope ratios for a large variety of consumers in intertidal and subtidal habitats, and their potential primary food sources [i.e., microphytobenthos (MPB), phytoplankton, and *Phragmites australis*] in a coastal bay system, Yeoja Bay of Korea, to test the hypothesis that the transfer of intertidal MPB-derived organic carbon to the subtidal food web can be mediated by motile consumers. Compared to a narrow δ^13^C range (−18 to −16‰) of offshore consumers, a broad δ^13^C range (−18 to −12‰) of both intertidal and subtidal consumers indicated that ^13^C-enriched sources of organic matter are an important trophic source to coastal consumers. In the intertidal areas, δ^13^C of most consumers overlapped with or was ^13^C-enriched relative to MPB. Despite the scarcity of MPB in the subtidal, highly motile consumers in subtidal habitat had nearly identical δ^13^C range with many intertidal foragers (including crustaceans and fish), overlapping with the range of MPB. In contrast, δ^13^C values of many sedentary benthic invertebrates in the subtidal areas were similar to those of offshore consumers and more ^13^C-depleted than motile foragers, indicating high dependence on phytoplankton-derived carbon. The isotopic mixing model calculation confirms that the majority of motile consumers and also some of subtidal sedentary ones depend on intertidal MPB for more than a half of their tissue carbon. Finally, although further quantitative estimates are needed, these results suggest that direct foraging by motile consumers on intertidal areas, and thereby biological transport of MPB-derived organic carbon to the subtidal areas, may provide important trophic connection between intertidal production and the nearshore shallow subtidal food webs.

## Introduction

Close trophic connection between neighboring coastal habitats has long been recognized by exchange of materials across the coastal ecotone [1,2]. The net flux between the systems is determined by the magnitude and direction of transport of materials, providing a trophic subsidy from highly to less productive habitats [3,4]. These habitats show a dichotomy between the importer or the exporter in material flux, and the two extremes are able to coexist depending on types of materials (e.g. C export *vs*. N import) [1]. Materials can also flow from less to more productive habitats if the former one is located downstream. Provision of trophic subsidy across the coastal ecotone has been well exemplified by outwelling of the excess materials of salt marshes, mangrove swamps, and seagrass meadows to coastal sea, supporting its productivity [5,6].

Likewise, although tidal flats are generally known to receive detritus and plankton from the coastal sea, rivers, and salt marshes [7], tidal export of organic matter synthesized on bare intertidal bed is also accomplished. This tidal export of organic matter is often due to tide- and wind-induced resuspension and seaward transport of microphytobenthos (MPB), of which productivity and biomass are higher than those of phytoplankton on marsh tidal flats [8,9]. In this respect, although the presence of diatom film increases sediment stability and thereby suppresses resuspension of both sediments and diatoms [10–12], pennate diatoms can be easily resuspended at relatively weak current velocities (~10 cm s^−1^) on silty sediment [13–15]. The resuspension of MPB is dependent on current velocity, sediment type, and algal biomass, bioturbation and thereby sediment erosion [14–17]. In contrast, resuspended MPB can be deposited locally due to rapid settling coupled with resuspended sediments [12]. In particular, under meso- or micro-scale tidal cycle of low (~3 m) tidal amplitude like Yeoja Bay (southern coast of Korea), the dissimilarity in community composition between intertidal MPB and subtidal phytoplankton may imply low importance of physical transport of MPB from intertidal to subtidal areas [18,19]. The lack of active resuspension of MPB may make the role of MPB in subtidal areas still unclear. Accordingly, while the trophic importance of benthic microalgae to secondary production on marsh tidal flats and as part of phytoplankton even in the adjacent subtidal area is well-known [8,12,17,20], trophic pathways which lead MPB to be incorporated into shallow subtidal food webs remain relatively unclear.

A potential flow of MPB from intertidal to subtidal areas may be related to the consumption of MPB by migrating species which feed on intertidal areas, and then export the organic matter they fed on when they migrate back to the subtidal areas. Juvenile fish, crabs, and shrimp migrate with the tides and seasons between the two areas [21–23]. Enclosure experiments using cages clearly showed that migrating species, such as fish and crustaceans, to the intertidal flats prey on resident macrofauna [7,24,25]. Their migration to the subtidal after feeding represents an export of organic matter synthesized on the intertidal areas. This suggests the existence of consumer-mediated transport pathways of MPB-derived organic carbon to nearshore marine food webs. Numerous studies have shown trophic relationships between the intertidal and the shallow coastal ecosystems by predator enclosure and exclusion experiments [7] and stomach content analyses [24,25], highlighting an important role of intertidal flats as nursery ground for continental shelf organisms and feeding ground for offshore fish in mangroves. These methods display just a snapshot for recent feeding events and have difficulties in offering information on ultimate sources of dietary materials. Alternatively, stable isotope composition can provide direct evidence of potential sources of organic matter exploited by the consumers and also trophic transfer through food webs, representing a long-term dietary consumption.

Determination of isotopic composition has been widely applied to elucidate trophodynamics since animal tissue composition usually reflects those of its diet [26,27]. Carbon isotopic composition (expressed as δ^13^C values) of primary producers is conserved in animal tissues with only a slight modification ranging from 0.5‰ to 1.2‰ per trophic level. Different producers (i.e., C_3_ plants, C_4_ plants, microphytobenthos, phytoplankton, etc.) have distinctly different isotopic signatures; therefore, δ^13^C values can be used to identify the ultimate sources of organic matter [28–30]. In contrast, nitrogen isotopic composition (expressed as δ^15^N values) can be used to estimate trophic position of animals because their δ^15^N values are 2−4‰ higher than those of their diets [29–31]. A number of studies have demonstrated a large difference in δ^13^C values between MPB and phytoplankton, and suggested that ^13^C-enrichment in both intertidal and subtidal consumer tissues reflects the contribution of MPB which provide important trophic subsidy to benthic food web[32–34].

If migrating species transfer MPB-derived organic matter into subtidal food web after foraging on the intertidal areas, then intertidal and subtidal consumers from lower to higher trophic levels should have high δ^13^C values close to the range of MPB δ^13^C values. The present study tested this hypothesis by comparing stable isotope ratios of sedentary and motile consumers in both intertidal and nearshore shallow subtidal food webs.

Some authors recently suggested that ^13^C-enrichment in nearshore benthic suspension feeders may be explained by other factors such as selective feeding on ^13^C-enriched pelagic particles rather than the feeding on ^13^C-enriched benthic sources [35,36]. In addition, some authors explained that larger trophic fractionation factor (4‰ and ~2‰, respectively) than previously accepted fractionation factor (~0.8‰) may contribute more to ^13^C-enrichment in littoral suspension feeders than previously expected by feeding on resuspended MPB [35–37]. To overcome those isotopic ambiguities, the present study also examined seasonal variations of isotope ratios of an infaunal (ark shell *Scapharca subcrenata*) and an epifaunal suspension feeder (Pacific oyster *Crassostrea gigas*) raised in phytoplankton-dominated subtidal habitats for a year. We also determined isotope ratios of consumers collected offshore, in a phytoplankton-based ecosystem. These analyses allowed us to confirm isotope ratios of consumers which feed exclusively on phytoplankton-derived organic matter.

## Materials and Methods

### Study area

**Fig 1:**
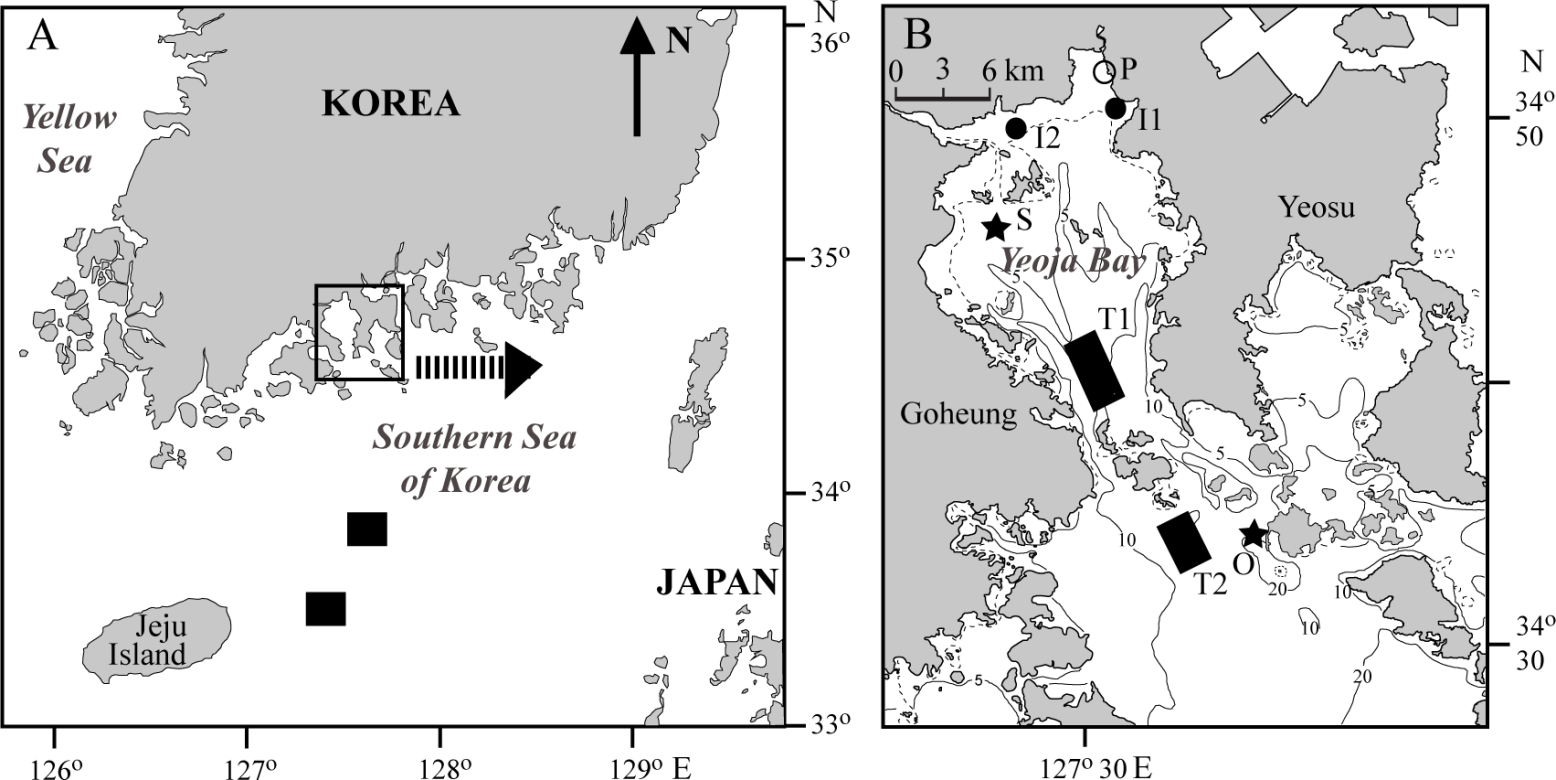
(A) Schematic map of the Southern Sea of Korea with offshore sampling locations and (B) two intertidal and two subtidal sampling sites in the Yeoja Bay. Black squares (A) and rectangles (B) represent offshore and subtidal (T1 and T2) trawling sites, respectively; black circles (B) indicate intertidal sites (I1 and I2) where fence nets were deployed. The common reed *Phragmites australis* was collected at Site P. The ark shell *Scapharca subcrenata* and the Pacific oyster *Crassostrea gigas* were cultured by local fishermen and collected at sites S and O, respectively.

Our study bay (Yeoja Bay) is located on the centre of the southern coast of the Korean peninsula (Fig 1). The bay extends roughly 30 km north from its mouth, and varies between 7.2 and 21.6 km from west (the Goheung Peninsula) to east (the Yeosu Peninsula). The bay is semi-enclosed, with a total area of about 320 km^2^, and relatively shallow, with a mean depth of 5.4 m. The tide is semidiurnal, with tidal amplitudes of approximately 3.6 m on spring tides and 2.3 m on neap tides. The bay contains broad (about 88.2 km^2^) and bare intertidal flats. The uppermost part of the intertidal flat is covered by broad (~7 km^2^) marsh plains of the common reed *Phragmites australis*. The sediment of the bay consist mostly of mud (∼0.5% sand, 30%–60% silt, 40%–70% clay; mean grain size of 1.3–4.5 μm) [18,19]. Freshwater flows into the bay mainly from the Dong, Isa and Beolgyo streams, located on the northern part of the bay. However, this freshwater discharge is generally low with a mean of 2.6 (± 1.1) m^3^ s^−1^ except for 14.2 and 10.5 m^3^ s^−1^ in July and August 2009, respectively [19]. Accordingly, although relatively low (~29) salinity is observed in the northern part of the bay during the rainy season because of the freshwater discharges from the streams, the salinity of the bay is between 31 and 34 [18]. Phytoplankton community in water column varies seasonally and is dominated by true phytoplankton species such as diatoms (*Skeletonema costatum*, *Eucampia zoodiacus*, *Chaetoceros* spp., *Thalassiothrix frauenfeldii*, *Coscinodiscus* spp., and *Nitzschia* spp.) and dinoflagellates (*Dinophysis* spp.) in most the bay area [18,19]. In contrast, MPB members on the intertidal mudflat are composed mainly of diatoms (*Pleurosigma* spp., *Amphora* spp., *Navicula* spp., and *Cylindrotheca closterium*) [this study]. Most of the intertidal and subtidal areas are free of macroalgae so that benthic algal primary production may depend mainly on intertidal microphytobenthos. Sedimentary chlorophyll *a* contents are much higher in intertidal areas (mean 5–44 μg g^−1^ dry sediment) than those in subtidal areas (0.34–0.93 μg g^−1^ dry sediment) [19,33].

The natural environments including extensive intertidal flat and common reed plain have been well conserved, allowing the tidal flat to be designated as a RAMSAR wetlands site of international importance in 2006. Macrobenthic community is well developed with high abundances (mean density of 2340 individuals m^−2^) of diverse species (over 270) [18,19]. Well conserved environments and possible high biodiversity of the bay provided the rationale for choosing the sampling sites of the present study.

The adjacent offshore area for isotopic comparison with the coastal bay system is open water area on the continental shelf (water depth = 97−102 m) of the southern sea of Korea (Fig 1). This area is predominated by the Tsushima Warm Current, which flows northeasterly as a branch of the Kuroshio, and has been thus considered to be a phytoplankton-based ecosystem [38,39].

### Ethics statement

There was no vertebrate or invertebrate species, which of experiment was required for Institutional Animal Care and Use Committees (IACUC) approval. Fishing and delivery of fish to laboratory was conducted by commercial fishermen, all fish were dead in the net when harvested, and there was no protected vertebrate or invertebrate species in our sample; IACUC approval for this sampling method was not required. Sample collection and export in the sampling area were permitted by Ministry of Oceans and Fisheries of Korea.

### Sample collection and preparation

#### Primary producers, suspended and sedimentary particulate organic matter

Samplings were conducted at two intertidal and two subtidal sites (∼10 m and 15−20 m depths of water) of the bay area and two offshore sites (Fig 1). The field work to collect animals was carried out in spring (April−May) and fall (September−October) 2009. Considering turnover time of animal tissues and the expected seasonal isotopic variation of organic matter sources, potential sources of organic matter, including suspended particulate organic matter (SPOM), MPB, and *Phragmites* marsh, were collected nearly at monthly intervals from February to November 2009.

Inshore SPOM was collected at the within-bay subtidal (Site S) and the outer-bay coastal (Site O) sites at midday high tide during spring tide periods, and offshore SPOM at the same sites with animal collection. For collection of SPOM at each site, approximately 20 l of seawater was collected using a van Dorn water sampler and prefiltered in situ with a 250-μm screen to remove large particles. The prefiltered water samples in the bay were transported to the laboratory as soon as possible. Considering the possibility of size-dependent variation in isotope ratios of SPOM due to different taxonomic composition [40], SPOM was divided into two fractions: coarse POM (CPOM, ≥ 20 μm) and fine POM (FPOM, < 20 μm). For this procedure, CPOM was filtered in situ with 20-μm mesh screen to determine isotope ratios of micro-size phytoplankton such as diatoms and then gathered into a test tube by scrapping. For collection of FPOM, the particulates in the prefiltered water were concentrated onto a precombusted (6 h, 550°C) Whatman GF/F filter in the laboratory. Filtered SPOM samples (both CPOM and FPOM) were divided into two parts and lyophilized before HCl treatment. Samples for δ^13^C measurements were acidified by adding several drops of 0.12 *N* HCl (for CPOM) or by fuming for about 5 h over concentrated HCl (for FPOM) to remove carbonates while samples for δ^15^N measurements were not acidified. For offshore SPOM samples, the above procedures were conducted on board. Then filters containing the SPOM for δ^13^C measurements were oven dried at 60°C for 48 h. MPB was collected by scraping the visible mat of benthic diatoms from the sediment surface of two intertidal sites (I1 and I2) during low tide. Sediment containing dense microalgal mats were spread to a depth of 2 cm in polystyrene trays and covered by a nylon screen of 63-μm mesh [41]. Then the screen was sheeted with thin layer (∼4 mm) of dried silica powder (ϕ 60–120 μm) and a filtered seawater was added. After extraction under daylight and table lamps at night, diatoms attached to the silica powder were washed out with milli-Q water, collected by centrifugation, and lyophilized. Sediment samples for isotopic analysis were collected from the upper 2 cm of the same sites where MPB were scrapped. These samples were kept frozen in sterile plastic bags with dry ice. Offshore sediment samples were collected using a gravity corer (Mooring Systems, Inc., MA). Slices of the surface sediments (top 2 cm) were separated from the corers. After thawing in the laboratory, carbonates were removed by treating sediment samples with 10% HCl solution until the bubbling stopped, oven dried at 60°C for 72 h, and homogenized by pulverizing with a mortar and pestle. Samples for nitrogen isotopic analysis of SOM were not acid-treated. Above ground parts of the common reed *Phragmites australis* were collected by hand at Site P, carefully cleaned to remove any epibionts, oven dried at 60°C for 72 h, and then ground into a fine powder with a mortar and pestle. These pretreated samples were kept frozen until subsequent analysis.

#### Oyster and ark shell

To capture the isotopic baseline of the pelagic food chain, the suspension-feeding bivalve, ark shell (*Scapharca subcrenata*), was bottom-cultured at the within-bay subtidal site (Site S) and oyster (*Crassostrea gigas*) was cultured in suspension at the depth of 2−6 m of the outer-bay coastal site (Site O) during a year cycle from February to November 2009. We expected that these two consumers can give an estimation of the isotopic values of the SPOM available where they were sampled. Those suspension feeders were monthly collected simultaneously with annual cycle of seston collection. Stable isotope ratios of the bivalve tissue were measured monthly for at least four individuals. All the bivalve specimens were transported to the laboratory and kept alive overnight in filtered seawater to evacuate their gut contents. After rinsing, they were carefully dissected and their whole bodies were prepared. The separated tissues were lyophilized and pulverized to a fine powder with a ball mill (Retsch MM200 Mixer Mill, Hyland Scientific, WA). Considering their low contents of lipids, defatting was skipped in the present study [42]. Their tissues were not acid treated. The pretreated bivalve samples were kept frozen until subsequent stable isotope measurements.

#### Consumers

On intertidal sites, sessile invertebrates were collected by hand or sieving sediments obtained by rectangular can corers (length = 305 mm, width = 205 mm, height = 200 mm) on a 1-mm mesh net at low tide. Motile fish and invertebrates were collected using a 1.2 m high and 100 m long oval-shaped fence net on the intertidal areas. The mesh size of fence net was 5 mm. The net was propped up with a wooden support at 3 to 4-m intervals on the sediment. When motile fauna migrated to the intertidal flats on the flood tide, they were trapped into a cod end on the lower part of the intertidal flat on the ebb tide and then collected at low tide. Specimens were sorted and invertebrates were kept alive overnight in filtered seawater from the sampling site to allow them to evacuate their gut contents, and then identified under a dissecting microscope in the laboratory. Only muscle tissues of live and intact mollusks, crabs and shrimps were collected to minimize contamination with other materials. Viscera of polychaetes were extracted by dissection and their remaining whole bodies were prepared. Amphipods were pooled to provide enough material for isotope analysis. While most of macro-invertebrate tissues were not acid treated, small invertebrates, including crustaceans, gastropods, and bivalves, were decalcified with 10% HCl solution until the bubbling stopped to remove probable effects of carbonates. These small invertebrate tissues for nitrogen isotopic analysis were not acid-treated. Fish were also dissected in the laboratory and only white muscle tissues of dorsal region were collected. In order to avoid effects of the species’ differences in the content of isotopically lighter lipids [43], fish tissue samples were defatted in a mixed (2:1:0.8) solution of methanol, chloroform and water [44]. Because lipid contents in most the invertebrate tissues have been known to be very low (< 5% of dry tissue weight in crabs, shrimp, cephalopods, and bivalves; < 2% in gastropods) compared to those (range 4−34%; > 10% in many cases) in fish [45], defatting of invertebrate tissues was skipped in the present study. The pretreated animal samples were also kept frozen until subsequent analysis.

At two subtidal (an inner- and another outer-bay site) and offshore sites, fish and invertebrates were collected using a bottom trawl. The trawl was 15 m long and 1.5 m high. Its opening was approximately 3 m. The mesh size of trawling net was 14 mm on the wings and belly of the net and 10 mm in the cod end. As mentioned above, a much smaller mesh net than commercial fishing gears (25−240 mm mesh net) was used to capture invertebrates. Trawling was conducted three times at each site for 0.5 h per each trawl set. Trawling speed was approximately 4 km h^−1^. Sediment samples for benthic invertebrates were collected using a 0.12 m^2^ van Veen grab. Benthic invertebrates collected by sieving sediments were cleaned of epibionts and sorted on board. After immediate transportation to the laboratory, invertebrates collected were kept alive overnight in filtered sea water. This procedure for offshore specimens was conducted on board. Subtidal and offshore specimens, and sediment slices were treated and prepared with the same manner as did for intertidal ones. After thawing, all taxa from each site were lyophilized and pulverized to a fine powder with a ball mill (Retsch MM200 Mixer Mill, Hyland Scientific, WA). Determination of the feeding modes of consumer species was based on the literature ([46–48]; http://www.fishbase.org/search.php; http://eol.org; http://www.uniprot.org).

### Stable isotope analyses

For measurements of carbon and nitrogen stable isotope abundances, 0.5 to 1.5 mg of powdered and homogenized samples were sealed in tin combustion cups and the whole body of the GF/F filter was wrapped with tin plate. Sealed samples were combusted at high temperature (1030°C) in an automated elemental analyzer (vario MICRO cube, Hanau, Germany). The resultant gases were then analyzed using an interfaced continuous-flow isotope ratio mass spectrometer (CF-IRMS; IsoPrime 100, Cheadle, U.K.). Isotopic values were expressed in delta (δ) notation as parts per thousand (‰) differences from the conventional standards (Vienna Pee Dee Belemnite and air N_2_ for carbon and nitrogen, respectively) according to the equation: δX = ([R_sample_/R_standard_] − 1) × 10^3^, where X is ^13^C or ^15^N and R is the ^13^C/^12^C or ^15^N/^14^N ratio, respectively (Fry and Sherr, 1984). International standards of sucrose (ANU C_12_H_22_O_11_; NIST, Gaithersburg, MD) and ammonium sulfate ([NH_4_]_2_SO_4_; NIST) were used and analyzed after every five samples to calibrate the system. The analytical reproducibility, based on the standard deviations of 20 replicates of urea, was approximately ≤ 0.15‰ for δ^13^C and ≤ 0.20‰ for δ^15^N.

### Statistical analysis

Multivariate analysis of variance (MANOVA) was used within the framework of a general linear model to examine if there were differences in dual isotope signatures (δ^13^C and δ^15^N values) among the organic matter sources (i.e. CPOM, FPOM, SOM, MPB, and *Phragmites australis*). Wilks’ lambda was used to evaluate the results. Normality was tested using the Shapiro-Wilk test (p < 0.05) and equal variance was determined using Levene’s test prior to MANOVA test. Appropriate means were compared using Tukey *post hoc* tests. Because we did not meet approximation of normality and equal variance after data transformation in isotope ratios of consumers, isotopic differences of consumers in the whole community level were compared using a Kruskal-Wallis test or a two-sample Kolmogorov−Smirnov test with habitats (intertidal, subtidal, and offshore) and seasons (spring and fall) as factors. These statistical procedures were performed using commercially available SPSS software (Chicago, USA). A hierarchical cluster analysis (Bray–Curtis similarity, average grouping methods) was conducted on the average δ^13^C and δ^15^N values of each species for all consumers collected from intertidal and subtidal areas in spring and summer. This test was conducted using a commercially available PRIMER software (Version 6, PRIMER-E, Plymouth, UK). Stable isotope ratios of cluster groups were compared using a permutational multivariate analysis of variance (PERMANOVA). Because δ^13^C and δ^15^N values of consumers in cluster groups were normally distributed and had equal variance, the isotopic differences among cluster groups were tested using the Tukey HSD multiple comparison test without prior transformation of isotope data. Further comparisons were made between individual taxa collected from intertidal and subtidal areas using two-sample t-test if necessary.

### Mixing model

We used an isotopic mixing model implemented in the software package SIAR (Stable Isotope Analysis in R) as Bayesian approach [49] to estimate relative contributions of CPOM, FPOM, SOM, and MPB to nutrition of higher-trophic-level consumers, and to identify major pathways organic matter derived from intertidal MPB. Input of isotopic values and trophic fractionation factors of consumers and their potential dietary sources is necessary to run the SIAR mixing model. This model takes into account the uncertainties of the input data and calculates the ranges of possible source contributions to each consumer. First, in order to determine the relative contributions of the above-mentioned basal resources to the nutrition of consumer groups, we tried to run the SIAR mixing model using δ^13^C and δ^15^N values of these sources and consumers. Trophic fractionation factors necessary to run the mixing model were determined by the values obtained from the present study for primary consumer groups (1.8 ± 0.2‰ for δ^13^C and 2.3 ± 0.7‰ for δ^15^N, respectively; see details in Results) and published for carnivorous (higher-trophic-level) species (1.3 ± 0.3‰ for δ^13^C and 3.3 ± 0.26‰ for δ^15^N, respectively) [30]. Second, in order to decipher trophic connection between intertidal flats and subtidal areas, we also tried to identify major pathways of organic matter derived from intertidal MPB. There is a clear discrimination in δ^13^C values between pelagic and benthic consumers in subtidal or continental shelf communities free of ^13^C-enriched benthic food sources [35,50,51]. They suggested that, although ^13^C-enrichment in benthic affinity components is determined by various biogeochemical processes, natural benthic ^13^C-enrichment can be used as an isotopic baseline to estimate the contribution of benthic affinity prey to the higher-level consumers. Therefore, we used subtidal pelagic and benthic primary consumers as isotopic baselines of the proxy of SPOM-feeding consumers and intertidal benthic deposit feeders as isotopic baseline of the proxy of MPB-feeding consumers, respectively, to calculate % contribution of each baseline prey to the nutrition of the higher-level consumers [52]. Relative dietary contributions of each basal resource or prey group to the high-trophic-level consumers were expressed by credibility intervals of 95% [49].

## Results

### Stable isotope ratios of major organic matter sources

**Fig 2:**
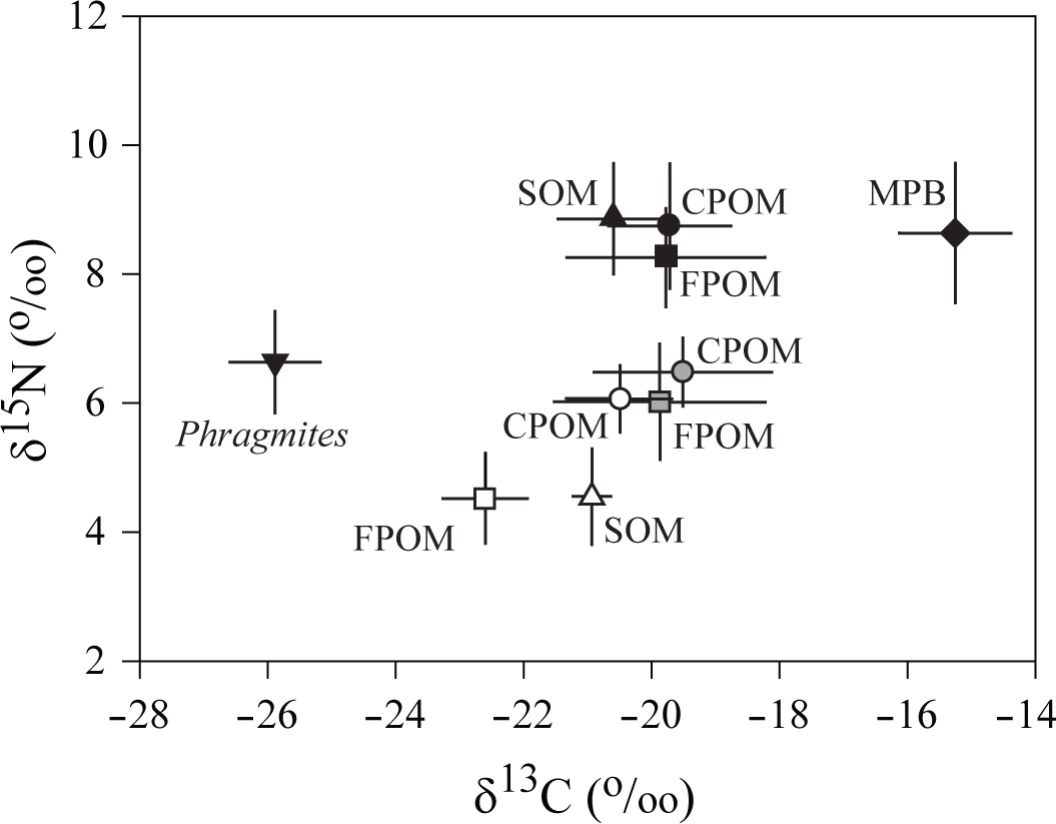
Mean of δ^13^C and δ^15^N values (±1 SD) of suspended particulate organic matter (POM), sedimentary organic matter (SOM), microphytobenthos (MPB), and the common reed *Phragmites australis*. Black, gray, and white colors for POM and SOM represent the within-bay (Site S), the outer-bay (Site O), and offshore sites, respectively. FPOM, fine POM fraction <20 μm; CPOM, coarse POM fraction of 20 to 180 μm.

**Fig 3:**
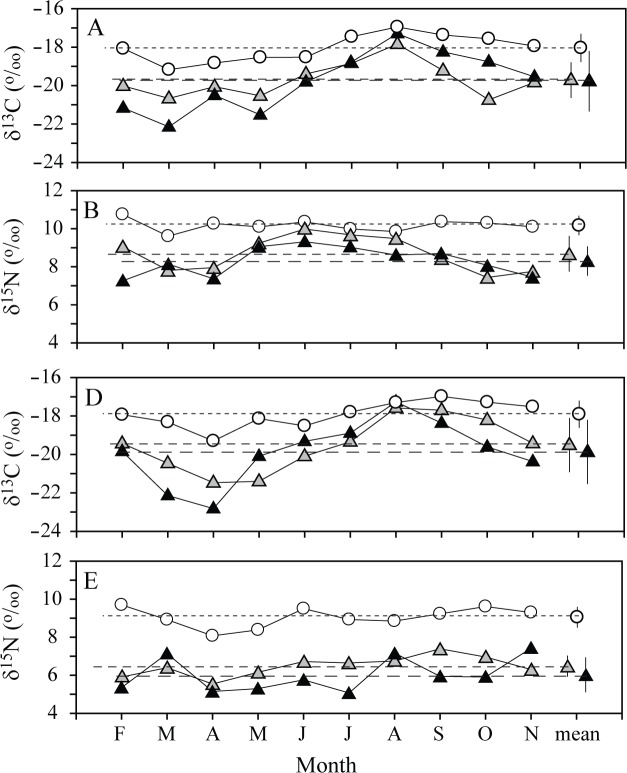
Seasonal variations in (A) δ^13^C and (B) δ^15^N values of *Scapharca subcrenata*, and (C) δ^13^C and (D) δ^15^N of *Crassostrea gigas*. Mean represents annual means (±1 SD). Circles represent the bivalves; triangles coarse (gray) and fine (black) particulate organic matter (CPOM and FPOM), respectively.

MANOVA test indicated significant differences in the isotopic signatures of the major primary sources of organic matter (CPOM, FPOM, SOM, MPB, and *Phragmites australis*; Wilks’ lambda = 0.031, *p* < 0.001; Fig 2). Results of ANOVA test indicated that this was due to differences in both δ^13^C and δ^15^N values (*F*_9, 130_ = 109.9 and *F*_9, 123_ = 51.9, *p* < 0.001 for both). Seasonal variability in their isotopic composition was slightly larger in δ^15^N values (CVs, 8.2–14.9%) than in δ^13^C (2.3–8.3%), and was largest in the outer-bay FPOM (Fig 3). Due to limited sampling seasons, isotopic variability of offshore SPOM was undetectable.

Despite such a considerable seasonal variability in inshore sources of organic matter, the means (± 1 SDs) and the ranges of δ^13^C values for each source overlap very little. No apparent seasonal trends in δ^13^C of MPB and *Phragmites* were found and their mean δ^13^C values held positions of both extremities of organic matter sources (Fig 2). MPB (mainly benthic diatoms) had the most enriched δ^13^C values among the measured sources of organic matter (Tukey’s HSD test, *p* < 0.05), representing a narrow range of −16.9 to −14.3‰ and an annual mean of −15.3 ± 0.9‰ (*n* = 19). In contrast, marsh vascular plant *Phragmites australis* had the lowest δ^13^C values (Tukey’s HSD test, *p* < 0.05), ranging from −27.4 to −24.8‰, with an annual mean of −25.9 ± 0.7‰ (*n* = 18). δ^13^C values of SPOM and SOM intervened between those of MPB and *Phragmites*. δ^13^C values of SPOM (both CPOM and FPOM) peaked in summer (August) both at the within- and the outer-bay sites (Fig 3). Although inshore FPOM had slightly broader ranges in δ^13^C values of −22.5 to −17.0‰ (mean −19.8 ± 1.5‰) and −22.8 to −17.3‰ (−19.9 ± 1.6‰) at the within- and the outer-bay sites, respectively, compared to −21.1 to −17.4‰ (−19.7 ± 1.0‰) and −21.5 to −17.6‰ (−19.5 ± 1.4‰) of CPOM, their annual means were nearly identical (Tukey’s HSD test, *p* = 0.706) with −19.7 ± 1.3‰ (*n* = 40) at the within-bay and −19.6 ± 1.5‰ (*n* = 20) at the outer-bay counterparts. Offshore CPOM and FPOM had similar mean δ^13^C of −20.5 ± 0.8‰ (*n* = 15) and −22.6 ± 0.7‰ (*n* = 14), respectively (Tukey’s HSD test, *p* = 0.123; Fig 2). The mean δ^13^C value of offshore FPOM was slightly more negative than those of inshore SPOM (Tukey’s HSD test, p < 0.05). The mean δ^13^C values of SOM were −20.6 ± 0.9‰ (*n* = 8) and −20.9 ± 0.3‰ (*n* = 6) at the within-bay and the offshore sites, respectively.

Mean δ^15^N values of the major sources of organic matter varied from 4.5 ± 0.7‰ to 8.7 ± 1.0‰. δ^15^N values of MPB ranged between 6.6 and 10.2‰, with an annual mean of 8.7 ± 1.1‰. CPOM and FPOM at the within-bay site had similar δ^15^N ranges of 7.4 to 10.2‰ (mean 8.7 ± 1.0‰) and 7.2 to 9.3‰ (8.3 ± 0.8‰), respectively, with mean values very close to that of MPB (Tukey’s HSD test, *p* = 0.990). However, their mean δ^15^N values were slightly higher than those at the outer-bay (6.5 ± 0.5‰ and 6.0 ± 0.9‰ for CPOM and FPOM, respectively) and offshore (6.1 ± 0.5‰ and 4.5 ± 0.7‰, respectively) sites (Tukey’s HSD test, *p* < 0.05). δ^15^N values of *Phragmites australis* ranged from 5.1 to 7.8‰, their mean of 6.6 ± 0.8‰ intervening between the within-bay and offshore SPOM. The mean δ^15^N values of 8.9 ± 0.9‰ and 4.6 ± 0.7‰ for SOM at the within-bay and offshore sites reflected a spatial difference in δ^15^N values of SPOM.

### Seasonal variation of isotopic signatures of the cultured bivalves

The δ^13^C and δ^15^N values of the ark shell (*Scapharca subcrenata*) and the oyster (*Crassostrea gigas*) showed similar seasonal fluctuations (CVs, 4.0 and 3.1% for ark shells and 3.9 and 5.8% for oysters) to those of SPOM in their habitats (Fig 3). They had similar δ^13^C ranges of −19.2 to −17.0‰ and −19.3 to −17.0‰, respectively, the values being slightly lower in spring than in summer-fall and peaking in August (*Scapharca subcrenata*) and September (*Cassostrea gigas*) when SPOM had the highest values. Their δ^15^N values varied from 9.6 to 10.4‰ and 8.1 to 9.7‰, respectively, without clear seasonal patterns in its fluctuation. While annual mean δ^13^C values were identical (Paired *t*-test, t_9_ = −1.116, p = 0.293) between *S*. *subcrenata* (−18.0 ± 0.7‰) and *C*. *gigas* (−17.9 ± 0.7‰), their annual mean δ^15^N values were about 1‰ higher (*t*_9_ = 7.602, *p* < 0.001) in the ark shells (10.2 ± 0.3‰) than in the oysters (9.1 ± 0.5‰). As a result, mean δ^13^C value of infaunal ark shells at the within-bay site was shifted from those of CPOM and FPOM by +1.7‰ and oysters cultured in suspension at the subsurface of the outer-bay site was by +1.6‰ and +2.0‰, respectively. Mean δ^15^N enrichments were observed by +1.5‰ and 1.9‰ in the ark shells and +2.6‰ and 3.0‰ in the oysters, respectively, compared to those of CPOM and FPOM.

### Stable isotope compositions of consumers

**Fig 4:**
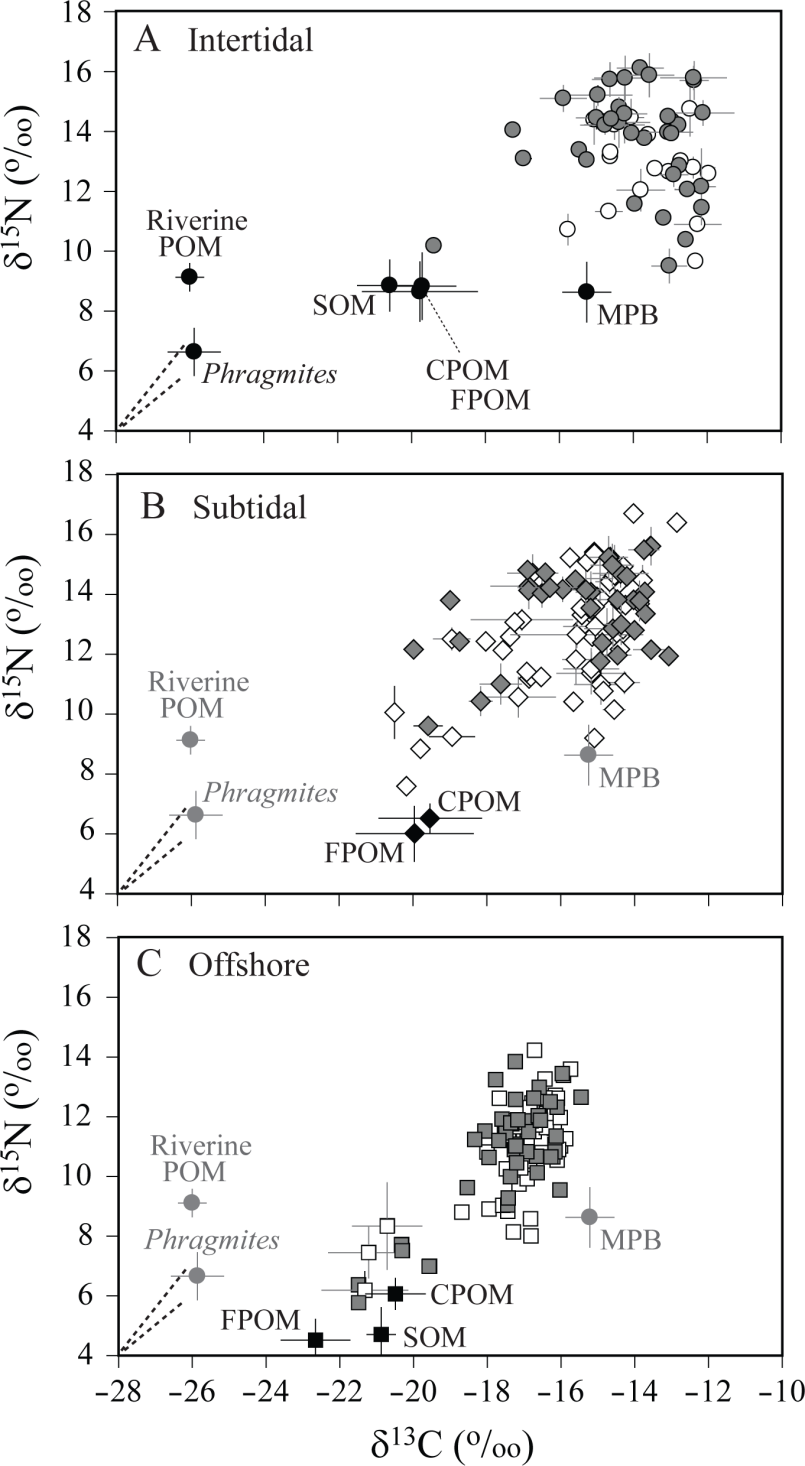
Dual plots of mean δ^13^C and δ^15^N values for food sources (black color) and consumers in spring (white) and fall (gray) in (A) intertidal, (B) subtidal, and (C) offshore habitats. Vertical and horizontal bars represent standard deviations. FPOM, fine (< 20 μm) suspended particulate organic matter; CPOM, coarse (> 20 μm) suspended particulate organic matter; SOM, sedimentary organic matter; MPB, microphytobenthos; *Phragmites*, *Phragmites australis*; Riverine POM, Riverine particulate organic matter [57]. MPB, *Phragmites*, and Riverine POM are in grey on B and C plots because they were not sampled at the subtidal and offshore areas. Dashed lines indicate the average trophic enrichment factors of +1.7‰ and +1.7‰ for δ^13^C and δ^15^N values in the ark shells; +1.8‰ and +2.8‰, respectively, in the oysters obtained in the present study.

**Fig 5:**
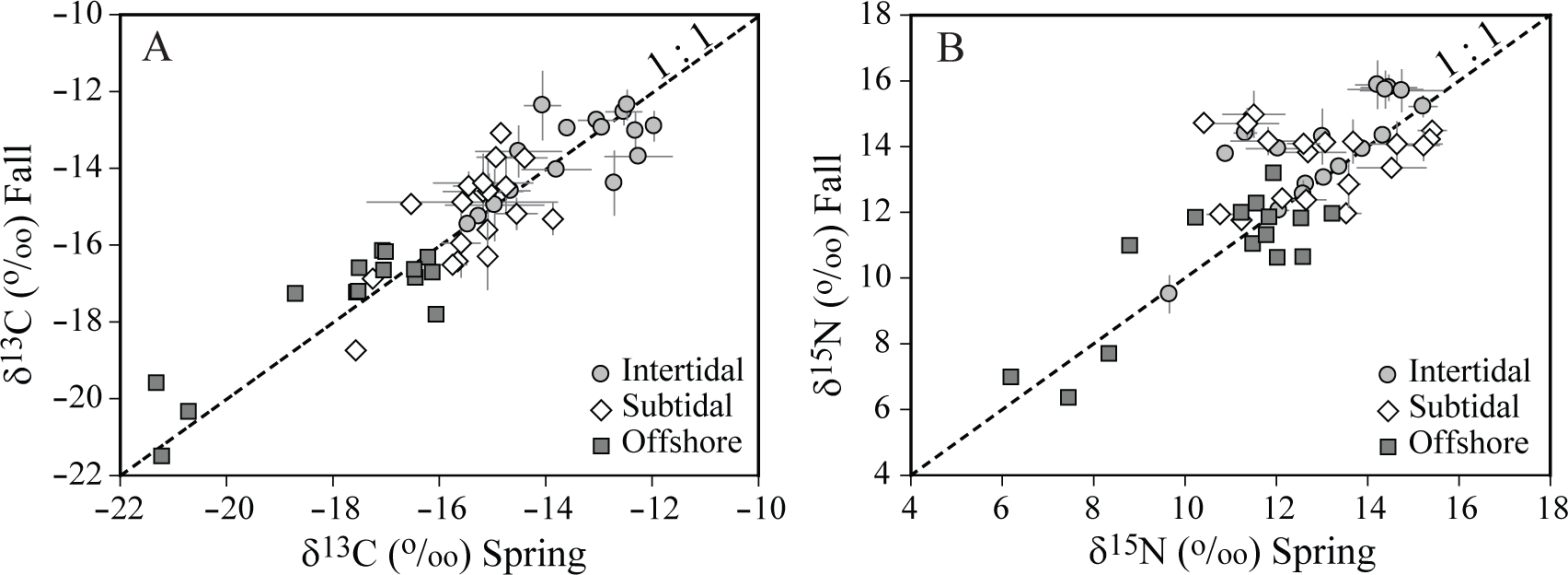
Scatter plots comparing (A) δ^13^C and (B) δ^15^N of consumers collected in both spring and fall in intertidal, subtidal, and offshore habitats. Values are mean δ^13^C and δ^15^N (‰ ± 1 SD). The dashed lines represent the 1:1 ratio line.

**Fig 6:**
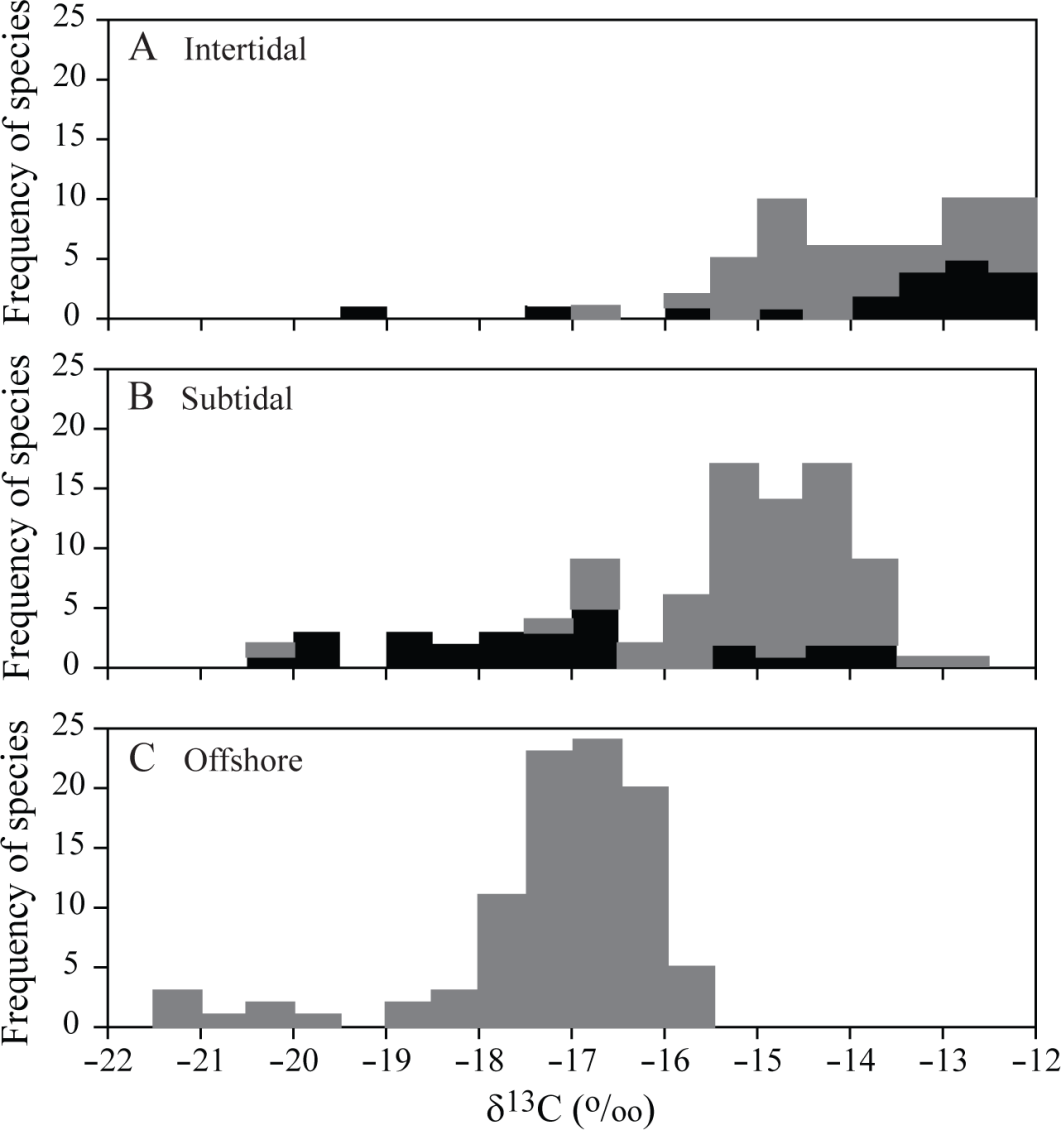
Frequency distribution of individual consumer δ^13^C (‰) collected in (A) intertidal, (B) subtidal, and (C) offshore habitats. Gray bar segments represent motile consumers and black bar segments sedentary consumers.

38, 74, and 77 consumer taxa were collected for the stable isotope analysis in the intertidal, subtidal, and offshore habitats, respectively (Table 1; S1 and S2 Appendices). The ranges of δ^13^C and δ^15^N values of consumers were nearly identical between spring (June) and fall (October) in all the three habitats, showing no distinct seasonal trends in consumer isotope composition of the entire community level of each habitat (Kolmogorov−Smirnov test, *p* = 0.985, 0.403, and 0.110 for δ^13^C; *p* = 0.121, 0.085, and 0.997 for δ^15^N in the three habitats, respectively; Fig 4). Indeed, with only the exception of δ^15^N values of omnivores and predators, there were no consistent and strong seasonal shifts in the δ^13^C and δ^15^N values of most taxa of consumers collected in both spring and fall in all three habitats (Fig 5). The pooled spring and fall δ^13^C values ranged from −20.4 to −12.0‰, −20.5 to −12.9‰, and −21.5 to −15.5‰ in the intertidal, subtidal, and offshore communities, respectively, and δ^15^N values ranged from 9.5 to 16.1‰, 7.6 to 16.7‰, and 5.8 to 14.2‰ in respective habitats (Fig 4). For the pooled spring and fall values, significant differences in both δ^13^C and δ^15^N values of consumers at the entire community level were detected among habitats (Kruskal−Wallis test, *p* < 0.001 for both cases). Subsequent two-sample Kolmogorov−Smirnov test revealed that the intertidal or subtidal communities had significantly enriched δ^13^C and δ^15^N values than the offshore community (*p* < 0.001 for both cases between intertidal or subtidal versus offshore). Further, while consumer δ^13^C values of the intertidal community were significantly enriched compared to the subtidal community (Kolmogorov−Smirnov test, *p* < 0.001), no difference in δ^15^N values of consumers was detected between intertidal and subtidal ones (*p* = 0.646). Frequency distributions in δ^13^C values of individual consumers displayed clear spatial distribution patterns that consumer δ^13^C values were more enriched in the intertidal community than those in the offshore community (Fig 6). The δ^13^C values of the subtidal consumers were slightly more variable (CV = 11.1%) than consumers of the intertidal and offshore habitats (10.5 and 6.9%, respectively), showing bimodal distribution.

**Table 1:** List of intertidal and subtidal consumers whose isotope composition was analyzed in the present study.

No	Taxon	No	Taxon	No	Taxon	No	Taxon
	**Intertidal**	27	*Acanthogobius flavimanu* (CA; E1/E2)		Gastropoda (sedentary)	43	*Amblychaeturichthys hexanema* (CA; E1/E1)
	Poychaeta (sedentary)	28	*Boleophthalmus pectinirostris* (CA; D/D)	16	*Aplysia* sp. (GR; B2/‒)	44	*Apogon lineatus* (CA; ‒/E1)
1	*Hediste japonica* (OM; ‒/B2)	29	*Engraulis japonicas* (CA; ‒/B2)	17	*Nassarius fortunei* (CA; B2/‒)	45	*Chelidonichthys spinosus* (CA; C1/C2)
2	*Perinereis vancaurica tetradentata* (OM; E1/E2)	30	*Konosirus puntatus* (CA; E1/E2)	18	*Nassarius* sp. (CA; ‒/E1)	46	*Cociella crocodile* (CA; E1/‒)
3	*A; E1/E1Perineris nuntia* (OM; C2/E1)	31	*Lateolabrax japonicas* (CA; E1/‒)	19	*Glossaulax didyma* (CA; E1/‒)	47	*Cryptocentrus filifer* (CA; E1/‒)
	Bivalvia (sedentary)	32	*Mugil cephalus* (CA; D/E1)	20	*Rapana venosa* (CA; E1/E1)	48	*Cynoglossus abbreviates* (CA; E1/‒)
4	*Crassostrea gigas* (SF; ‒/A)	33	*Muraenesox cinereus* (CA; ‒/E2)	21	*Zeuxis siquijorensis* (SC; ‒/B2)	49	*Cynoglossus joyneri* (CA; E1/‒)
5	*Moerella iridescens* (DF; ‒/D)	34	*Odontamblyopus lacepedii* (CA; E2/E2)		Crustacea (motile)	50	*Cynoglossus robustus* (CA; E1/‒)
	Cephalopoda (motile)	35	*Pholis nebulosa* (CA; ‒/E1)	22	*Alpheus japonicus* (OM; ‒/E1)	51	*Cynoglossus* sp. (CA; ‒/E1)
6	*Octopus minor* (CA; ‒/E1)	36	*Takifugu niphobles* (CA; E1/E1)	23	Gammaridian amphipods (DF; A/‒)	52	*Decapterus maruadsi* (CA; C1/‒)
	Gastropoda (sedentary)	37	*Takifugu* sp. (CA; ‒/E2)	24	*Arcania undecimspinosa* (CA; ‒/D)	53	*Engraulis japonicas* (CA; C1/‒)
7	*Batillaria multiformis* (DF; D/D)	38	*Thryssa kammalensis* (CA; E1/E1)	25	*Charybdis bimaculata* (CA; C2/‒)	54	*Inimicus japonicas* (CA; ‒/E2)
8	*Bullacta exarata* (DF; D/D)		**Subtidal**	26	*Charybdis japonica* (CA; E1/‒)	55	*Konosirus punctatus* (CA; ‒/E1)
9	*Cerithidea largillierti* (DF; ‒/D)		Hydrozoa (motile)	27	*Lysmata vittata* (CA; E2/‒)	56	*Larimichthys polyactis* (CA; E1/E1)
10	*Cerithidea ornata* (DF; D/‒)	1	*Aurelia aurita* (CA; B/‒)	28	*Metapenaeus joyneri* (CA; E1/E1)	57	*Leiognathus nuchalis* (CA; C2/B2)
11	*Cerithideopsilla djadjariensis* (DF; ‒/D)		Echiura (sedentary)	29	*Oratosquilla aratoria* (CA; C2/E1)	58	*Mugil cephalus* (CA; E1/‒)
12	*Littorina brevicula* (DF; D/‒)	2	*Urechis unicinctus* (DF; E1/‒)	30	*Pagurus* sp. (OM; C2/B2)	59	*Odontamblyopus lacepedii* (CA; ‒/E2)
13	*Lunatia gilva* (CA; D/E2)		Polychaeta (sedentary)	31	*Palaemon gravieri* (CA; C2/‒)	60	*Pampus argenteus* (CA; E1/E1)
14	*Zeuxis siquijorensis* (SC; E2/E2)	3	*Glycera* sp. (CA; ‒/B2)	32	*Parapenaeopsis tenella* (CA; E1/E1)	61	*Paralichthys olivaceus* (CA; B2/‒)
	Crustacea (motile)	4	*Lepidonotus* sp. (CA; B2/B2)	33	*Philyra pisum* (CA; C2/‒)	62	*Pennahia argentata* (CA; E1/‒)
15	*Alpheus japonicus* (OM; E2/E2)	5	*Nereis* sp. (OM; B2/‒)	34	*Philyra* sp. (CA; ‒/D)	63	*Platycephalus indicus* (CA; E1/E1)
16	*Callianassa japonica* (DF; ‒/E2)	6	*Lumbrineris japonica* (OM; B2/B1)	35	*Portunus trituberculatus* (CA; E1/E1)	64	*Pleuronichthys cornutus* (CA; ‒/E1)
17	*Charybdis japonica* (CA; ‒/E1)	7	*Sternapsis scutata* (DF; C1/‒)	36	*Trachysalambria curvirostris* (CA; E1/‒)	65	*Pseudoblennius cottoides* (CA; E2/‒)
18	*Cleistostoma dilatatum* (DF; ‒/E2)		Bivalvia (sedentary)	37	Mixed zooplankton (SF; A/‒)	66	*Repomucenus sagitta* (CA; E1/‒)
19	*Exopalaemon carinicauda* (CA; E1/E2)	8	*Atrina pectinata* (SF; ‒/C1)		Stelloridea (sedentary)	67	*Sardinella zunasi* (CA; E1/‒)
20	*Helice tridens* (CA; ‒/E1)	9	*Crassostrea gigas* (SF; ‒/A)	38	*Asterias amurensis* (OM; ‒/B1)	68	*Scomber japonicas* (CA; C2/E1)
21	*Hemigrapsus penicillatus* (OM; C2/E1)	10	*Fulvia mutica* (SF; A/‒)		Echinodea (sedentary)	69	*Sebastes inermis* (CA; E1/‒)
22	*Ilyoplax pusilla* (DF; ‒/D)	11	*Moerella* sp. (DF; C1/‒)	39	*Hemicentrotus* sp. (OM; ‒/B1)	70	*Sillago sihama* (CA; *C2/*‒)
23	*Macrophthalmus japonicus* (DF; ‒/D)	12	*Musculus senhausia* (SF; A/‒)		Holothuroidea (sedentary)	71	*Takifugu* sp. (CA; E1/‒)
24	*Metapenaeus joyneri* (CA; E1/E1)	13	*Scapharca subcrenata* (SF; ‒/C1)	40	*Protankyra bidentata* (DF; E1/E1)	72	*Thryssa adelae* (CA; E1/B2)
25	*Pachygrapsus crassipes* (OM; ‒/D)		Cephalopoda (motile)		Fish (motile)	73	*Thryssa kammalensis* (CA; E1/B2)
26	*Philyra pisum* (SC; E1/D)	14	*Sepia* sp. (CA; E1/E1)	41	*Acanthogobius flavimanus* (CA; E2/‒)	74	*Upeneus japonicas* (CA; E1/‒)
	Fish (motile)	15	*Sepiella* sp. (CA; E1/‒)	42	*Acanthopagrus schlegeli* (CA; C2/‒)		

**Fig 7:**
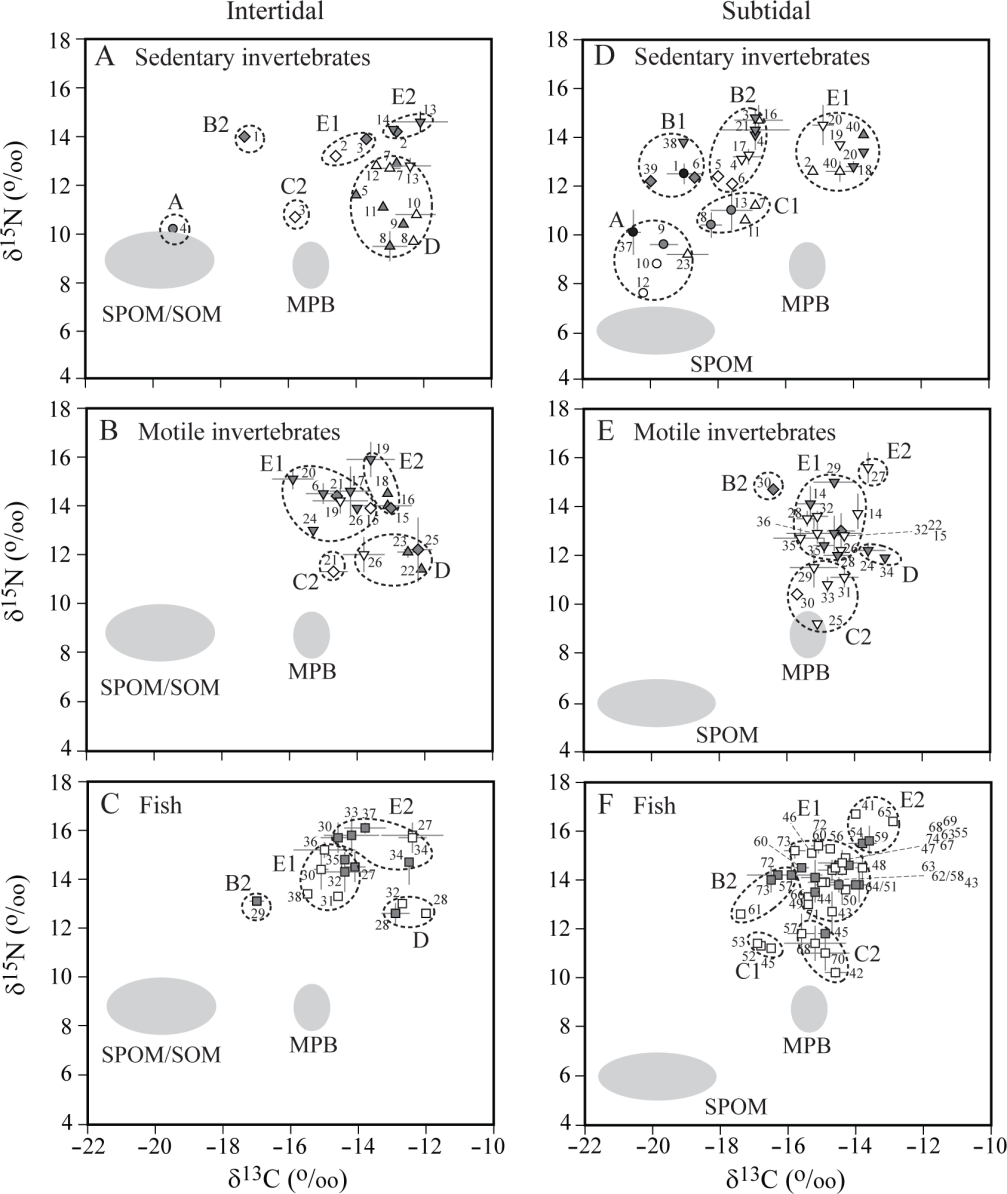
Dual plots of mean δ^13^C and δ^15^N values for sedentary and motile invertebrates, and fish in spring (white) and fall (gray) in (A−C) intertidal and (D−F) subtidal habitats. The groups of taxa were clustered according to the result of hierarchical cluster analysis (see details in Methods). Numbers designate different taxa (see Table 1 for species code). Circle, suspension feeder; triangle, deposit feeder; diamond, omnivore; inverted triangle, carnivore and scavenger; square, fish. Grey ellipses represent potential sources of organic matter: SPOM, suspended particulate organic matter; SOM, sedimentary organic matter; MPB, microphytobenthos.

Feeding modes and cluster groups (spring/fall) are given in parenthesis: SF, suspension feeder; DF, deposit feeder; GR, grazer; OM, omnivore; CA, carnivore; SC, scavenger. The codes of cluster group of consumers are given in Fig 7.

### Identification of trophic groups

Based on average δ^13^C and δ^15^N values for all consumers collected from both intertidal and subtidal areas in spring and summer, cluster analysis showed that they were classified into five main groups (at 93% similarity level) and three groups of these classifications were divided into two subgroups (Fig 7). Subsequent PERMANOVA test revealed that isotopic signatures of consumers differed significantly among subgroups (Pseudo-*F*_7, 143_ = 83.172, *p* = 0.001). Furthermore, PERMANOVA test revealed that grouping of the consumers were related to both their mobility (Pseudo-*F*_1, 149_ = 11.53, *p* = 0.001) and feeding mode (Pseudo-*F*_4, 146_ = 16.063, *p* = 0.001). The Tukey HSD test (*p* = 0.05 level) revealed a clear separation in δ^13^C between sedentary (and very few motile) invertebrates (Group A, Subgroups B1, B2, and C1) and motile consumers (both invertebrates and fish; Group D, Subgroups E1, E2, and C2) in the subtidal. Group A consisted of suspension-feeding bivalves with pelagic zooplankton and gammaridian amphipods, and showed the most negative δ^13^C values (mean ± 1SD: −19.7 ± 0.6‰) and the lowest δ^15^N values (9.3 ± 0.9‰) of the consumer groups, falling within the δ^13^C and δ^15^N ranges of SPOM. Subgroup B1, comprising subtidal omnivorous sea urchin and polychaete, and carnivorous seastar and jellyfish, had consistent δ^13^C values (−18.9 ± 1.0‰) with but higher δ^15^N values (12.7 ± 0.6‰) than those of Group A. Subgroup B2 included some omnivores and carnivores, and five fish species, and Subgroup C1 two infaunal suspension-feeding bivalves, two deposit feeders, and three fish species. They were collected mostly from the subtidal and had slightly more positive δ^13^C values (−17.0 ± 0.7‰ and −17.2 ± 0.6‰, respectively) compared to those of species in Group A and Subgroup B1. While δ^15^N of Subgroup B2 (13.7 ± 0.8‰) was higher than that of Subgroup C1 (11.0 ± 0.4‰), their δ^13^C values overlapped each other. As a result, with the exception of Subgroup E1 (including three carnivorous gastropods, echiura, and deposit-feeding sea cucumber) that had close δ^13^C values to MPB, the majority of subtidal sedentary invertebrates (Group A, Subgroups B1, B2, and C1) constituted consumer members that had more negative δ^13^C than consumers of another δ^13^C mode in Fig 6B. Their δ^13^C range overlapped with that of the offshore consumers (Fig 6).

In contrast, Group D had the most enriched δ^13^C values (mean: −12.8 ± 0.6‰) and was comprised mostly of intertidal sedentary deposit feeders and some motile crabs and fish. Their δ^15^N values (11.8 ± 1.0‰) overlapped with Subgroups C1 and C2. Subgroup E1 consisted mainly of motile carnivorous invertebrates and fish collected from both intertidal and subtidal areas, displaying δ^13^C values (−14.9 ± 0.6‰) corresponding to the δ^13^C range of MPB. Similar δ^13^C values (−15.1 ± 0.5‰) were found in Subgroup C2, which contained carnivorous invertebrates and fish mainly in the subtidal. Their δ^15^N values were on average 3‰ higher in Subgroup E1 (13.9 ± 0.9‰) than that (10.9 ± 0.7‰) in Subgroup C2. Subgroup E2 had the most enriched δ^13^C (−13.2 ± 0.7‰) and the highest δ^15^N values (15.2 ± 0.9‰) of the consumer groups, consisting of two carnivorous gastropods in the intertidal and motile shrimps, crab, and some fish in both intertidal and subtidal areas. Accordingly, the major groups of motile invertebrate and fish had a consistent δ^13^C range between the intertidal and subtidal, and close δ^13^C values to those of most intertidal sedentary invertebrates. These latter groups (Group D, Subgroups E1, E2, and C2) accounted for the consumers that had less negative δ^13^C values than sedentary invertebrates in the subtidal, as shown in Fig 6B.

### Bayesian mixing model calculation

**Fig 8:**
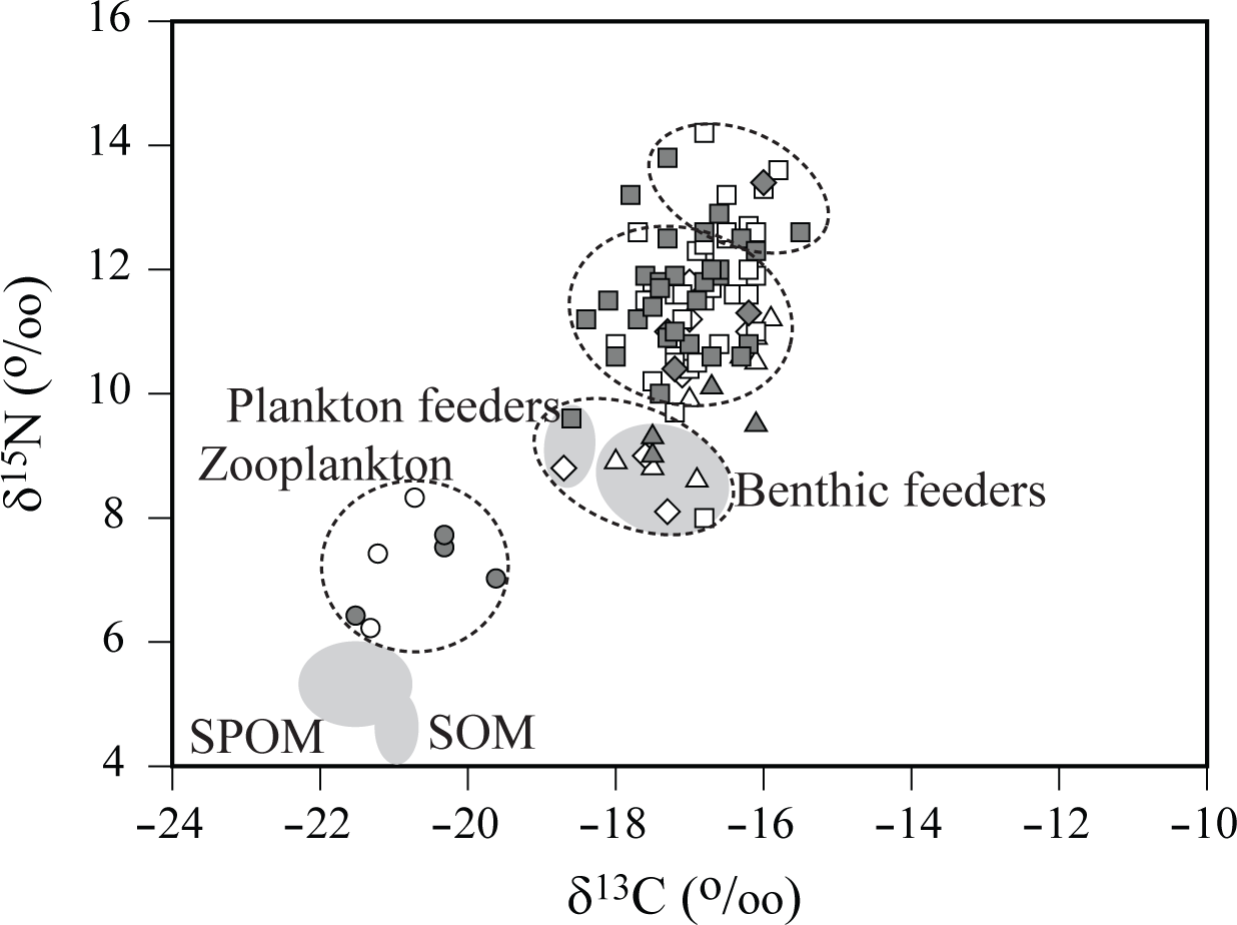
Dual plots of mean δ^13^C and δ^15^N values for zooplankton, other invertebrates, and fish in spring (white) and fall (gray) in offshore habitats. The groups of taxa were clustered according to the result of hierarchical cluster analysis (see details in Methods). Circle, zooplankton; triangle, arthropods; diamond, mollusks; square, fish. Grey ellipses represent suspended particulate organic matter (SPOM) and sedimentary organic matter (SOM). Species name and isotope data are given in S1 Appendix.

Zooplankton and sedentary primary consumers in the subtidal area were divided into two groups (Groups A and C1 in Fig 7) mainly by their δ^13^C values. A similar result was also observed in isotopic signatures of the offshore consumers (Fig 8). Bayesian mixing model calculation for the relative contribution of the basal food sources (i.e. CPOM, FPOM, SOM, and MPB) to consumer production revealed that they constituted equally the dietary source supporting both intertidal and subtidal pelagic feeders (Group A; 7–18% to 25–43%) of primary consumers (Table 2). CPOM, SOM, and MPB displayed considerable contribution (25–43%, 5–21%, and 36–43%, respectively) to the nutrition of subtidal benthic feeders (Group C1). In contrast, MPB was estimated to be the major nutritional source (84–94%) for Group D, comprised mostly of intertidal sedentary deposit feeders. In cases of higher-trophic-level consumers, while the above-mentioned basal food sources were estimated to contribute 15–34% to 20–39% (Group B1) and 4–10% to 42–64% (Group B2) to the nutrition of subtidal sedentary consumers, MPB contributed 40–46% and 51–54% (Group E1), and 74–80% and 62–72% (group E2) to the nutrition of motile consumers in both intertidal and subtidal areas, respectively.

**Table 2:** The 95% credibility intervals of trophic contribution (%) of organic matter sources or prey groups to the diet of the consumer groups.

Consumers		Habitat	Organic matter source				Prey groups		
Cluster/Main trophic groups			CPOM	FPOM	SOM	MPB	Inter-/subtidal pelagic-affinity prey (A)	Subtidal benthic-affinity prey (C1)	Intertidal benthic prey (D)
Lower trophic level									
A	Pelagic feeders	Intertidal	16–36	16–36	17–36	15–34			
		Subtidal	25–43	8–29	24–41	7–18			
C1	Benthic feeders	Subtidal	25–43	0–8	5–21	36–43			
D	Benthic feeders	Intertidal	0–5	0–3	0–3	84–94			
Higher trophic level									
B1	Sedentary consumers	Subtidal	17–36	15–34	20–39	16–33	40–59	30–51	0–9
B2	Sedentary consumers	Subtidal	9–28	3–20	42–64	4–10	36–47	33–48	12–23
E1	Motile consumers	Intertidal	9–29	7–24	2–17	40–46	44–58	5–21	34–36
		Subtidal	1–8	1–4	32–40	51–54	35–42	20–28	36–40
E2	Motile consumers	Intertidal	1–9	0–6	1–7	74–80	15–31	21–39	34–36
		Subtidal	1–12	1–10	1–9	62–72	8–20	31–49	38–51

The codes of cluster group of consumers are given in Table 1 and Fig 7. CPOM, FPOM, SOM and MPB stand for coarse, fine suspended particulate organic matter, sedimentary organic matter and microphytobenthos, respectively.

To identify major pathways organic matter derived from intertidal MPB, the δ^13^C and δ^15^N values of Groups A, C1, and D were then chosen as isotopic baselines of pelagic- and benthic-affinity prey and intertidal benthic prey organisms. Using these isotopic baselines, SIAR mixing-model calculation revealed that both pelagic affinity prey (Group A) and subtidal benthic-affinity prey (Group C1) contribute significantly to the diets of Groups B1, B2, E1, and E2 (8–20% to 40–59% and 5–21% to 30–51%, respectively). In contrast, intertidal benthic prey (Group D) was estimated to play an important role as dietary contributors to both intertidal and subtidal motile consumers (34–36% and 36–40% for Group E1; 34–36% and 38–51% for Group E2, respectively), but its contributions to the nutritional source of Groups B1 and B2 appeared to be very little (0–9% and 12–23%, respectively).

## Discussion

In subtidal areas where MPB is abundant in sediments or resuspended MPB is transported from intertidal areas due to strong tidal currents, it has been widely demonstrated that MPB can provide an important trophic subsidy to coastal food webs [32,34,52,53]. This assumption was based on much more ^13^C-enrichment in tissues of the consumers feeding on MPB than those depending their dietary source on pelagic phytoplankton. The ^13^C-enrichment in consumer tissues has been also observed in subtidal systems that are colonized by abundant MPB or macroalgae, reflecting the heavy δ^13^C values of those benthic micro- or macroalgae [52, 54]. As mentioned earlier, the subtidal area of Yeoja Bay can be characterized by low abundances of benthic micro- and macroalgae [19,33]. Accordingly, the source of the ^13^C-enriched values in consumers in subtidal ecosystem of Yeoja Bay is relatively equivocal, because it is unexpected that the consumers feed on benthic primary producers [c.f. 35,36]. Our stable isotope data indicate that MPB-derived materials may be significant in the subtidal food web of Yeoja Bay. Indeed, subtidal motile consumers had higher δ^13^C values than those in subtidal sedentary and offshore consumers that are considered to feed exclusively on phytoplankton-derived materials. A consistency in δ^13^C values between intertidal consumers and subtidal motile ones further confirms trophic connection between intertidal and nearshore shallow subtidal food web.

### Potential food sources

As generally found in other intertidal flats, MPB accounts for one of the most important primary producers on broad and bare intertidal flat in Yeoja Bay. Its biomass amounts to an annual mean of 35.7 (± 22.9) mg chlorophyll *a* m^−2^ in the top 20 mm of the sediments [55]. Similar amounts of pheopigments *a* (a chlorophyll *a* degradation product) are also found with annual means of 17.5 ± 8.4 mg m^−2^ in pheophytins *a* and 22.7 ± 18.7 mg m^−2^ in pheophorbides *a*. It appears that outwelling of intertidal MPB toward subtidal area (i.e. seaward transport of resuspended MPB) is not as great as those observed in other coastal seas (e.g. the Ems estuary [8]; Ariake Sound [34,53]). There are several lines of evidence on low availability of MPB in the subtidal sediments and SPOM of Yeoja Bay. Both intertidal and subtidal sediments of Yeoja Bay are composed of mostly mud and chlorophyll *a* content of surface sediment was generally much higher in intertidal than in subtidal areas [19,33,55]. Although chlorophyll *a* concentrations are highly variable from 1.7 μg l^–1^ (December) to 11.5 μg l^–1^ (August) in subtidal water column [42], the abundance of pennate benthic diatoms in subtidal water column is very low [18,19]. Furthermore, relatively high content (annual mean = 1.27 μg g^−1^) of phaeopigments compared to chlorophyll *a* content (annual mean = 0.40 μg g^−1^) in subtidal sediments suggests a predominance of phytodetritus in the muddy sediments [19]. Such a low abundance of MPB may be explained by the lack of light reaching the bottom sediment. Indeed, long-term regular monitoring results showed that the water of Yeoja Bay can be characterized by low transparency [56,57]. The observed Secchi depths fell within very narrow ranges of 0.5−1.3 m (annual mean ± 1SD, 0.8 ± 0.3 m) and 0.7−2.5 m (1.3 ± 0.6 m) all the year round in both 2011 and 2013, respectively. As estimated by the Secchi depths, shallower euphotic depth (mean 2.2 m and 3.5 m, respectively) than the mean water depth (5.4 m) of the bay may restrict light penetration to the bottom. Such a low transparency of water may account for the above-mentioned low chlorophyll *a* content in subtidal sediments and the scarcity of local input (turnover by both production and biomass) of MPB in the studied subtidal.

Assuming that isotope values of SPOM and SOM represent mixtures of potential organic matter sources, their δ^13^C values (~ –20‰) in the study sites would reflect a mixture of marsh vascular plants (*Phragmites* –25.9‰; *Suaeda japonica* –29.2‰ [58]), riverine POM (~ –26‰ [58]), and benthic diatoms (–15.3‰). However, our previous study reported that a contribution of ^13^C-depleted carbon sources (i.e. marsh plants and riverine POM) is limited to the SPOM and SOM pools of supralittoral marsh habitats and their contribution to the intertidal and subtidal POM pools is minimal [58]. Then, if SPOM and SOM represent a mixture of MPB and phytoplankton (or phytodetritus), they would have more ^13^C-enriched values than our SPOM and SOM values (~ –20‰), which is typical of marine phytoplankton [28] and has been generally found for SPOM in coastal waters free of benthic producers of the Korean peninsula [33,40]. As observed by photosynthetic pigment composition during the present study period, the chemotaxonomical determination showed that diatoms were the most dominant group of phytoplankton in Yeoja Bay all the year round, accounting for 73.2% (± 9.7, September) to 84.9% (± 7.6, August) of chlorophyll *a* with the exception of October and November [unpubl. data]. Although cryptomonads constituted a considerable fraction in October and November (38.4 ± 11.1% and 34.0 ± 6.5% of total abundance, respectively), diatoms still formed a dominant part (24.6 ± 4.9% and 47.3 ± 8.1%, respectively) during that time. This indicates that pelagic diatoms can contribute a large part to subtidal SPOM that is ^13^C-depleted compared to benthic microalgae. Accordingly, consistency in δ^13^C between SPOM and SOM indicated that the contribution of MPB-derived carbon to the subtidal SPOM and SOM pools of Yeoja Bay is minor, as also speculated by the lack of pennate diatoms. Further δ^13^C consistency between intertidal SPOM (mean −20.5‰ and −19.4‰) and SOM (−20.6‰ and −19.4‰ [58]), subtidal and offshore SPOM and SOM concludes that SPOM from all these areas is dominated by phytoplankton (i.e. low influence of MPB) and that SOM composition is also dominated by phytodetritus. The effect of terrestrial organic matter on the composition of SOM is known to be minimal even in the intertidal area [58]. Indeed, our SPOM collection at high tides may explain a reduced contribution of resuspended MPB to the water column organic mixture. However, along with the consistency in δ^13^C values between SPOM and SOM, δ^13^C values of suspension feeders (the ark shell and the oyster) may guarantee the low proportion of physical transport (resuspension and advection) of MPB from the intertidal to the subtidal system. Moreover, although intertidal oysters (*Crassostrea gigas*) were collected once in October, their δ^13^C value (−19.4‰) was very close to that of subtidal oysters (–19.6‰) and SPOM, but much more ^13^C-depleted than –12.8‰ of deposit feeders in the same site (Fig 6). Considering that the intertidal oysters were collected about 1 m above surface sediment on the wooden support of fence net, this result may be indicative of a lack of strong resuspension of MPB even on the intertidal flat of the bay [59].

### Stable isotope signatures of basal food resources and consumers

δ^13^C ranges of potential sources of organic matter (i.e. primary producers and riverine POM [58]) in Yeoja Bay and the adjacent offshore areas were well comparable to those previously reported for temperate estuarine and coastal systems worldwide as well as other coasts of the Korean peninsula [33,58,60, references therein]. Overall δ^13^C range of consumers (−21.5 to −15.5‰) collected from inshore and offshore was very far from that (−27.4 to −24.8‰) of marsh vascular plants and riverine POM on a δ^13^C−δ^15^N isospace but overlapped with or was more ^13^C-enriched than the range of SPOM and MPB (Fig 3), indicating an important contribution of the latter ^13^C-enriched sources to the consumers’ diets. Organic matter derived from marsh vascular plants (such as *Phragmites*) and river discharge, though less nutritious, can be incorporated into the coastal food web as their detritus is available [61–63]. However, the minor contribution of ^13^C-depleted vascular plant or river-borne materials to consumer diets may reflect their minimal availability in SPOM and SOM pools of the study area as explained earlier.

As observed in δ^13^C values of offshore consumers (Fig 8), a larger difference (~4‰) in δ^13^C values was also found between Groups A-B1 versus Groups C1-B2 of subtidal sedentary invertebrates (Figs 6 and 7). The latter groups were more ^13^C-enriched than expected by the general trophic enrichment factor (~1.8‰). More positive δ^13^C values (up to 4‰) in benthic feeders than in pelagic feeders have been generally observed in continental shelf and slope food webs [50,64,65]. The elevated δ^13^C of the latter groups may conform to a combination of several possible factors including: a seasonal pulse of heavy carbon to benthic feeders, selective feeding of suspension feeders on ^13^C-enriched material of the POM pool, feeding of deposit feeders on refractory or recycled organic matter of the SOM pool, and benthic versus pelagic feeding of secondary consumers [35,51,66]. The δ^13^C values of the cultured bivalves were slightly elevated in summer (August and September). Such a ^13^C-enrichment in their tissues probably reflected a transient increase in δ^13^C values of SPOM [67]. Despite such transiently elevated δ^13^C values, their annual δ^13^C ranges suggest that further ^13^C-enrichment in the inshore consumers is unlikely to be attributable to a seasonal pulse of heavy carbon or their selective feeding on ^13^C-enriched material of the POM pool. Consistent δ^13^C ranges between the ark shell and the oyster, and slightly high δ^15^N values of the ark shell compared to that of the oyster reflected δ^13^C and δ^15^N values in their respective habitat within and outside the bay. In addition, an increase in δ^13^C of the cultured bivalves compared to those of SPOM was well consistent with the previous finding [37] and an increase in their δ^15^N values also fell within the range of generally accepted trophic fractionation [29,30]. This result indicates that they feed exclusively on SPOM in their habitats. Interestingly, motile consumers had more positive δ^13^C values than those of the majority of sedentary consumers in the subtidal habitat. Because motile consumers are generally more opportunistic or/even carnivorous and thus they occupy a higher trophic level than non-motile feeders, their δ^13^C values may reflect partly the trophic fractionation (i.e. ^13^C-enrichment). However, further increase in δ^13^C values of subtidal motile invertebrates and fish cannot be accounted for by the above-mentioned enrichment processes and needs additional interpretation in relation to an important contribution of ^13^C-enriched benthic primary producers to their nutrition. MPB is regarded as the most possible candidate that can explain their ^13^C-enrichment in the present study, as examined below according to trophic transfer through diverse feeding groups and their mobility.

### Carbon transfer from primary producers to primary consumers

Suspension feeders and deposit feeders were characterized as primary consumers, based on their low δ^15^N values, relative to other consumers. Based on their δ^13^C values and feeding modes, these primary consumers are separated into three major functional groups. The first group (Group A) clusters the epifaunal suspension-feeding bivalves (*Crassostrea gigas* and *Musculus senhausia*), mixed zooplankton, and suprabenthic amphipods. This suspension-feeder group, which accounts for the lowest trophic position of the subtidal food web, has consistent δ^13^C values with SPOM and SOM. Considering a dominance of phytodetritus in the SOM pool, this result indicats that their nutrition is directly dependent on pelagic sources of organic matter (25–43% CPOM, 8–29% FPOM, and 24–41% SOM). Their low δ^15^N values close to that of SPOM have been often observed in some suspension feeders, confirming the previous finding that they can utilize particles selected on the basis of either nutritional quality or different size classes [40,51,65]. This explanation may be also supported by seasonal proximity in δ^15^N values between SPOM and suspension feeders (*S*. *subcrenata* and *C*. *gigas*) in the present study (Fig 3). The second group (Subgroup C1) includes infaunal suspension-feeding bivalves (*Atrina pectinata* and *Scapharca subcrenata*) and deposit feeders (*Sternapsis scutata* and *Moerella* sp.) mainly in the subtidal area, suggesting that although they employ different feeding strategies, they utilize the same nutritional source (25–43% CPOM, 0–8% FPOM, 5–21% SOM, and 36–43% MPB) on the sediment surface [51]. Their δ^13^C values are more positive than those of the water-column suspension feeders, indicating the consumption of deposited organic matter that could be ^13^C-enriched during the degradation and bacterial recycling processes as discussed earlier. ^13^C-enrichment in benthic food chain in comparison to the pelagic counterpart appears to be a general feature in both inshore and offshore of the study area. Such a δ^13^C variation between primary consumer groups allows us to identify benthic versus pelagic pathways in trophic transfer of organic matter originated from pelagic production [50,51]. Lastly, the third primary consumer group (Group D) is composed mainly of intertidal deposit feeders. More positive δ^13^C values of these consumers than that of MPB indicate that they feed directly on MPB (84–94%) as their dietary source in intertidal areas. Exclusive utilization of MPB by the deposit-feeding bivalves (e.g. *Moerella iridescens*), gastropods (e.g. *Batillaria multiformis* and *Bullacta exarata*) and crabs (*Ilyoplax pusilla* and *Macrophthalmus japonicus*) on intertidal sediment surface has been well demonstrated [33,68]. Isotopic signatures of intertidal fish such as mudskipper (*Boleophthalmus pectinirostris*) and gray mullet (*Mugil cephalus*) were clustered into this group, reflecting their selective feeding on the mudflat MPB [69,70]. It has been generally known that these primary consumers can integrate the temporal variation in isotopic signatures of their dietary sources [31] and, furthermore, some can reflect their exploitation with respect to the degradation or microbial recycling status of POM [65,71]. According to their broad δ^13^C range (–20.5 to –12.0‰), these primary consumer groups can provide isotopic baselines to track main trophic pathways through which organic matter derived from pelagic versus benthic production is transferred to the higher-level consumers.

### Trophic pathways to higher-level consumers and MPB-derived carbon transport

Significantly higher δ^15^N values of carnivores and the majority of fish (Groups B1, B2, E1, and E2) than those of primary consumers indicate that they feed at higher trophic levels. In contrast, omnivores showed a large δ^15^N range that spans primary and secondary consumer groups, reflecting their wide dietary spectrum and thereby vertical expansions of trophic niches [71]. With the exception of a few species (e.g. suspension-feeding oyster *C*. *gigas*, omnivorous rag worm *Hediste japonica*, and planktivorous anchovy *Engraulis japonica*), higher-level trophic consumers in intertidal have a narrower range of δ^13^C values (–15.9 to –12.1‰) that are very close to those (–14.0 to –12.9‰) of deposit-feeder group. This suggests that the majority of both sedentary and motile consumers in the intertidal areas depends a considerable part of their nutrition on MPB-derived organic matter, and thus MPB are the predominant basal resources in the intertidal areas. In contrast, the range of δ^13^C values (–20.0 to –12.1‰) of the secondary or omnivorous consumers is as wide as that of primary consumers in the subtidal area, suggesting subtidal consumers exploit different mixtures of microalgal production (i.e. either benthic or pelagic, or both). A significant difference in δ^13^C signatures was found between sedentary and motile consumers at higher-trophic levels as observed in primary consumers. Indeed, the SIAR mixing-model results indicated that this isotopic variation reflects differential inputs of SPOM- versus MPB-derived organic matter in their diets (Table 2).

Some non-motile invertebrates (Subgroup B1 including sea urchin, polychaete, seastar, and jellyfish) had the close correspondence in δ^13^C to suspension feeder-zooplankton (Group A) and higher δ^15^N (on average 3.4‰) than the latter group in subtidal, suggesting that they take in their basal nutrition from phytoplankton-derived organic matter through this prey-predator relationship between them. Indeed, the mixing model estimated their dependence on pelagic and benthic affinity prey to be 40−59% and 30−51%, respectively. Of these invertebrates, the predatory jellyfish *Aurelia aurita* utilize largely copepod and microzooplankton prey [72]. The sea urchin *Hemicentrotus* sp., despite a preference for seaweeds, shows omnivorous feeding behavior on remnant of dead animals or plants at low algal level, or cannibalism in starving condition [73]. The seastar *Asterias amurensis* has a preference for bivalve molluscs [74]. Accordingly, exclusive utilization of phytoplankton-derived organic matter by these consumers is likely to reflect their omnivorous and carnivorous feeding on pelagic-affinity prey. Another non-motile carnivorous group had slightly more positive δ^13^C than but consistent δ^15^N with the above-mentioned group, indicating a close trophic relationship with benthic prey group (Group C including infaunal suspension feeders and deposit feeders). The mean estimated contribution of pelagic and benthic affinity prey to the nutrition of these sedentary consumers in subtidal area was 36−47% and 33−48%, respectively. This result suggests that phytoplankton-derived organic matter is the primary nutritional source for those that sequestrate their food through both pelagic and benthic affinity prey. Seasonally broad δ^13^C range and slightly low δ^15^N values of nereid worms (*Hediste* sp. and *Lumbrineris japonica*) compared to other carnivores seem to reflect their diverse feeding characteristics from suspension- and deposit- to carnivorous feeding [46]. Benthic prey utilization by carnivores of this group may be also explained by their known feeding characteristics such as carnivorous habit of polychaete *Glycera* sp. and *Lepidonotus* sp. preying on small polychaetes, amphipods, and organic debris [46], clam-eating of moon snail *Nassarius fortunei* [75], and scavenging of burned nassa *Zeuxis siquijorensis* [76]. Interestingly, a close correlation (δ^15^N = 0.99 * δ^13^C + 29.82; *n* = 32, *r* ^2^ = 0.45, *p* < 0.001) between δ^13^C and δ^15^N values is found in these two non-motile carnivorous groups and the related primary consumer groups (including suspension feeders and deposit feeders) in subtidal area, pointing out their simple and linear relationship with phytoplankton-derived organic matter.

Because of the scarcity of MPB in the studied subtidal area, it may be assumed that they make little contribution to the POM pool and to whole subtidal food web. In contrast to this assumption, stable isotope signatures of motile consumers indicate that MPB-derived organic matter may be significant in the subtidal food web of Yeoja Bay. The majority of motile consumers (Subgroup E1 including mostly shrimps, crabs, and fish) in both intertidal and subtidal areas fell within the same narrow δ^13^C range (−15.8 to −13.8‰), which is more positive than that of subtidal sedentary consumers (−20.5 to −16.9‰) and close to that of intertidal deposit feeders (−14.0 to −12.1‰). Their δ^15^N values were on average 2.1 to 4.6‰ higher than those of primary consumer groups, supporting the idea that their δ^13^C signatures may integrate their feeding history in both subtidal and intertidal areas. The mixing model calculation confirms a consistent dependence on both intertidal deposit-feeding group (36−40%) and subtidal pelagic affinity prey (35−42%), suggesting their indirect dependence on MPB and phytoplankton via multiple pathways. Another motile consumer group (Subgroup E2 including mostly fish) had slightly higher δ^13^C (on average 1.9‰) and δ^15^N (1.3‰) values than those of the above-mentioned Subgroup E1, displaying also high reliance on intertidal deposit feeders (38−51%) with considerable reliance on subtidal benthic affinity prey (31−49%). This suggests the increased importance of MPB to their basal nutritional source in both intertidal and subtidal areas. In addition, the isotopic difference between Subgroups E1 and E2 is likely to reflect their different use of subtidal pelagic versus benthic affinity prey as revealed by the mixing-model estimates.

Unexpectedly low δ^15^N values compared to other motile consumers were found in some consumers (Subgroup C2 including shrimps, crabs, and fish). However, their δ^13^C range (−15.7 to −14.6‰) was consistent with that of other motile consumers, suggesting their considerable ingestion on prey items that contain MPB-derived organic matter. One of the likely explanations for their low δ^15^N values is that they migrate recently to inshore after recruited offshore and probably reflect previous ingestion on offshore prey that has significantly lower δ^15^N values than inshore one. Likewise, similar δ^15^N values but slightly more negative δ^13^C values of three fish species, *Chelidonichthys spinosus*, *Decapterus maruadsi*, and *Engraulis japonicas*, which are included Subgroup C1, compared to the above-mentioned Subgroup C2 could be also explained by their immediate joining inshore food web network.

Dense assemblages of various fish and crustacean species from juveniles to adults are immigrated with the flood tide on the intertidal flat of Yeoja Bay (J. S. Hong, Inha Univ., unpublished data), as reported for neighboring tidal flats [21–24]. Historically, commercial fishing efforts by fishermen have long been made for these tidal migrants, including fish, crabs, and shrimps, using fence net in the bay. A consistency in δ^13^C values between sedentary and motile consumers in intertidal flat confirms the previous gut-content and cage-experiment findings that these tidal migrants have high foraging activity on intertidal ^13^C-enriched prey [7,24,25]. Further, a consistency in δ^13^C range between inter- and subtidal motile consumers supports the interpretation that MPB-originated ^13^C-enriched organic matter can be incorporated into the subtidal food web by foraging of motile consumers on the intertidal areas and subsequent migration to the subtidal areas. High δ^13^C values in a few carnivorous snails and whelks (sedentary invertebrates of Subgroup E1) of the subtidal are likely to be linked by this type of incorporation of ^13^C-enriched organic matter. As a result, our investigation reveals a linear trophic pathway that phytoplankton-derived organic matter is transferred through pelagic and benthic affinity prey to higher-level consumers in subtidal habitat [64,66]. Furthermore, our results also provide isotopic evidence that motile consumers, such as fish, crabs and shrimps, may integrate both intertidal and subtidal production, mediating close trophic connection between intertidal and nearshore shallow subtidal food web at a whole-bay scale. Our isotopic evidence suggests the importance of biological rather than physical transport as a main transport pathway of the ^13^C-enriched intertidal MPB to the subtidal food web. Further study to provide quantitative estimates of trophic subsidies and to validate trophic importance of resuspened MPB in this bay system will be needed to support this conclusion.

## Supporting Information

S1 Appendix Stable isotope ratios (‰; mean ± SD) of consumers collected from the intertidal and subtidal habitats in Yeoja Bay of the Korean peninsula. (DOC)

S2 Appendix Stable isotope ratios (‰) of consumers collected from offshore sites in the southern coast of the Korean peninsula. All samples were pooled by each taxon (zooplankton) or species (other consumers).
(DOC)
