# Supplementary material for: Linking Intertidal and Subtidal Food Webs: Consumer-Mediated Transport of Intertidal Benthic Microalgal Carbon
Source: PLoS One. 2015 Oct 8;10(10):e0139802. doi: 10.1371/journal.pone.0139802 (PMC4598165; doi:10.1371/journal.pone.0139802)
Supplement: S1 Appendix — (DOC) [file pone.0139802.s001.doc]

| Species name | Spring | |  | | | | Fall | | |
| --- | --- | --- | --- | --- | --- | --- | --- | --- | --- |
|  | δ^13^C | δ^15^N | | n |  | δ^13^C | | δ^15^N | n |
| Intertidal |  |  | |  |  |  | |  |  |
| Annelida |  |  | |  |  |  | |  |  |
| Polychaeta |  |  | |  |  |  | |  |  |
| *Hediste japonica* (Rag worm) |  |  | |  |  | −17.3 | | 14.0 | 1 |
| *Perinereis vancaurica tetradentata* (Nereid worm) | −14.6 | 13.2 | | 1 |  | −12.8 | | 14.2 | 1 |
| *Perineris nuntia* (Nereid worm) | −15.8 ± 0.1 | 10.7 ± 0.5 | | 2 |  | −13.7 | | 13.8 | 1 |
| Mollusca |  |  | |  |  |  | |  |  |
| Bivalvia |  |  | |  |  |  | |  |  |
| *Crassostrea gigas* (Pacific oyster) |  |  | |  |  | −19.4 | | 10.2 | 1 |
| *Moerella iridescens* (Tellinid clam) |  |  | |  |  | −14.0 | | 11.6 | 1 |
| Cephalopoda |  |  | |  |  |  | |  |  |
| *Octopus minor* (Long arm octopus) |  |  | |  |  | −15.0 ± 0.5 | | 14.5 ± 0.4 | 3 |
| Gastropoda |  |  | |  |  |  | |  |  |
| *Batillaria multiformis* (Mud creeper) | −13.0 ± 0.3 | 12.7 ± 0.1 | | 3 |  | −12.8 | | 12.9 | 1 |
| *Bullacta exarata* (White bubble shell) | −12.3 ± 0.3 | 9.7 ± 0.2 | | 2 |  | −13.0 ± 0.5 | | 9.5 ± 0.6 | 2 |
| *Cerithidea largillierti* (Banded horn sanil) |  |  | |  |  | −12.6 | | 10.4 | 1 |
| *Cerithidea ornata* (Banded horn sanil) | −12.3 ± 0.6 | 10.9 ± 0.1 | | 2 |  |  | |  |  |
| *Cerithideopsilla djadjariensis* (Banded horn sanil) |  |  | |  |  | −13.2 | | 11.1 | 1 |
| *Littorina brevicula* (Korean common periwinkle) | −13.4 | 12.8 | | 1 |  |  | |  |  |
| *Lunatia gilva* (Fortune's moon snail) | −12.4 ± 0.6 | 12.8 ± 0.3 | | 3 |  | −12.1 ± 0.8 | | 14.6 ± 0.4 | 4 |
| *Zeuxis siquijorensis* (Burned nassa) | −12.9 ± 0.1 | 14.3 ± 0.1 | | 2 |  | −12.9 ± 0.1 | | 14.3 ± 0.1 | 2 |
| Arthropoda |  |  | |  |  |  | |  |  |
| Crustacea |  |  | |  |  |  | |  |  |
| *Alpheus japonicus* (Japanese snapping shrimp) | −13.6 | 13.9 | | 1 |  | −13.0 | | 13.9 | 1 |
| *Callianassa japonica* (Japanese ghost shrimp) |  |  | |  |  | −13.1 ± 0.7 | | 14.0 ± 0.2 | 2 |
| *Charybdis japonica* (Japanese swimming crab) |  |  | |  |  | −14.2 ± 0.6 | | 14.6 ± 1.0 | 4 |
| *Cleistostoma dilatatum* (Manicure ghost crab) |  |  | |  |  | −13.1 | | 14.5 | 1 |
| *Exopalaemon carinicauda* (Ridgetail prawn) | −14.5 ± 0.8 | 14.2 ± 0.5 | | 6 |  | −13.6 ± 0.7 | | 15.9 ± 0.7 | 3 |
| *Helice tridens* (Three-spined shore crab) |  |  | |  |  | −15.9 ± 0.6 | | 15.1 ± 0.4 | 2 |
| *Hemigrapsus penicillatus* (Japanese shore crab) | −14.7 ± 0.4 | 11.3 ± 0.2 | | 3 |  | −14.6 | | 14.4 | 1 |
| *Ilyoplax pusilla* (Pea ghost crab) |  |  | |  |  | −12.1 | | 11.4 | 1 |
| *Macrophthalmus japonicus* (Japanese ghost crab) | −12.5 ± 0.3 | 12.1 ± 0.2 | | 2 |  | −12.5 ± 0.3 | | 12.1 ± 0.2 | 2 |
| *Metapenaeus joyneri* (Shiba shrimp) | −15.3 ± 0.1 | 13.0 ± 0.1 | | 3 |  | −14.7 ± 0.5 | | 12.1 ± 0.3 | 3 |
| *Pachygrapsus crassipes* (Striped shore crab) |  |  | |  |  | −12.2 ± 0.4 | | 12.2 ± 1.3 | 4 |
| *Philyra pisum* (Pea pebble crab) | −13.8 ± 0.6 | 12.0 ± 0.7 | | 2 |  | −14.0 | | 13.9 | 1 |
| Fish |  |  | |  |  |  | |  |  |
| *Acanthogobius flavimanus* (Genuine goby) | −14.1 ± 0.3 | 14.5 ± 0.6 | | 15 |  | −12.4 ± 0.9 | | 15.8 ± 0.4 | 10 |
| *Boleophthalmus pectinirostris* (Mudskipper) | −12.0 | 12.6 | | 1 |  | −12.9 ± 0.4 | | 12.6 ± 0.4 | 3 |
| *Engraulis japonicus* (Anchovy) |  |  | |  |  | −17.0 ± 0.2 | | 13.1 ± 0.0 | 2 |
| *Konosirus puntatus* (Dotted gizzard shad) | −15.1 ± 0.2 | 14.4 ± 0.8 | | 2 |  | −14.6 ± 0.5 | | 15.7 ± 0.6 | 4 |
| *Lateolabrax japonicus* (Sea bass) | −14.6 | 13.3 | | 1 |  |  | |  |  |
| *Mugil cephalus* (Grey mullet) | −12.7 | 13.0 | | 1 |  | −14.4 ± 0.8 | | 14.3 ± 0.8 | 6 |
| *Muraenesox cinereus* (Conger pike) |  |  | |  |  | −14.2 ± 0.8 | | 15.8 ± 0.7 | 2 |
| *Odontamblyopus lacepedii* (Green eel goby) | −12.5 ± 0.1 | 14.7 ± 0.9 | | 2 |  | −12.4 ± 0.4 | | 15.7 ± 0.6 | 5 |
| *Pholis nebulosa* (Tidepool gunnel) |  |  | |  |  | −14.4 | | 14.8 | 1 |
| *Takifugu niphobles* (Grass puffer) | −15.0 ± 0.9 | 15.2 ± 0.3 | | 4 |  | −15.0 ± 0.9 | | 15.2 ± 0.3 | 4 |
| *Takifugu* sp. (Puffer) |  |  | |  |  | −13.8 ± 0.6 | | 16.1 ± 0.2 | 2 |
| *Thryssa kammalensis* (Anchovy) | −15.5 | 13.4 | | 1 |  | −15.5 | | 13.4 | 1 |
|  |  |  | |  |  |  | |  |  |
| Subtidal |  |  | |  |  |  | |  |  |
| Cnidaria |  |  | |  |  |  | |  |  |
| Hydrozoa |  |  | |  |  |  | |  |  |
| *Aurelia aurita* (Jellyfish) | −19.0 ± 0.5 | 12.5 ± 0.4 | | 2 |  |  | |  |  |
| Annelida |  |  | |  |  |  | |  |  |
| Echiura |  |  | |  |  |  | |  |  |
| *Urechis unicinctus* | −15.2 | 12.6 | | 1 |  |  | |  |  |
| Polychaeta |  |  | |  |  |  | |  |  |
| *Glycera* sp. (Glycerid worm) |  |  | |  |  | −16.9 | | 14.8 | 1 |
| *Lepidonotus* sp. (Scale worm) | −17.3 | 13.1 | | 1 |  | −16.9 | | 14.1 | 1 |
| *Hediste* sp. (Rag worm) | −18.0 | 12.4 | | 1 |  |  | |  |  |
| *Lumbrineris japonica* (Nereid worm) | −17.6 | 12.1 | | 1 |  | −18.7 | | 12.4 | 1 |
| *Sternapsis scutata* (Scutate stermaspid worm) | −16.9 | 11.2 | | 1 |  |  | |  |  |
| Mollusca |  |  | |  |  |  | |  |  |
| Bivalvia |  |  | |  |  |  | |  |  |
| *Atrina pectinata* (Korean common penshell) |  |  | |  |  | −18.2 ± 0.3 | | 10.4 ± 0.5 | 3 |
| *Crassostrea gigas* (Pacific oyster) |  |  | |  |  | −19.6 ± 0.4 | | 9.6 ± 0.1 | 2 |
| *Fulvia mutica* (Egg cockle) | −19.8 | 8.8 | | 1 |  |  | |  |  |
| *Moerella* sp. (Tellinid clam) | −17.2 | 10.6 | | 1 |  |  | |  |  |
| *Musculus senhausia* (Green mussel) | −20.2 | 7.6 | |  |  |  | |  |  |
| *Scapharca subcrenata* (Ark shell) |  |  | |  |  | −17.6 ± 0.6 | | 11.0 ± 0.7 | 8 |
| Cephalopoda |  |  | |  |  |  | |  |  |
| *Sepia* sp. (Cuttlefish) | −13.9 | 13.7 | | 1 |  | −15.3 ± 0.4 | | 14.1 ± 0.7 | 5 |
| *Sepiella* sp. (Cuttlefish) | −14.3 ± 0.2 | 12.8 ± 0.1 | | 2 |  |  | |  |  |
| Gastropoda |  |  | |  |  |  | |  |  |
| *Aplysia* sp. (Sea hare) | −16.8 ± 0.7 | 14.7 ± 0.6 | | 2 |  |  | |  |  |
| *Nassarius fortunei* (Moon snail) | −17.1 ± 0.4 | 13.2 ± 0.3 | | 4 |  |  | |  |  |
| *Nassarius* sp. (Moon snail) |  |  | |  |  | −14.0 ± 0.2 | | 12.8 ± 0.2 | 2 |
| *Glossaulax didyma* (Real bladder moon snail) | −14.4 | 13.7 | | 1 |  |  | |  |  |
| *Rapana venosa* (Purple whelk) | −14.9 ± 0.3 | 14.5 ± 0.8 | | 2 |  | −13.7 | | 13.4 | 1 |
| *Zeuxis siquijorensis* (Burned nassa) |  |  | |  |  | −16.9 ± 1.0 | | 14.3 ± 0.8 | 2 |
| Arthropoda |  |  | |  |  |  | |  |  |
| Crustacea |  |  | |  |  |  | |  |  |
| *Alpheus japonicus* (Japanese snapping shrimp) |  |  | |  |  | −14.4 ± 0.4 | | 13.0 ± 0.7 | 2 |
| Gammaridian amphipods | −18.9 ± 0.6 | 9.2 ± 0.1 | | 2 |  |  | |  |  |
| *Arcania undecimspinosa* (Eleven-spined pebble crab) |  |  | |  |  | −13.6 ± 0.5 | | 12.2 ± 0.3 | 2 |
| *Charybdis bimaculata* (Two-spot swimming crab) | −15.1 | 9.2 | | 1 |  |  | |  |  |
| *Charybdis japonica* (Japanese swimming crab) | −14.4 ± 0.1 | 12.2 ± 0.1 | | 2 |  |  | |  |  |
| *Lysmata vittata* (Indian lined shrimp) | −13.6 | 15.6 | | 1 |  |  | |  |  |
| *Metapenaeus joyneri* (Shiba shrimp) | −15.4 ± 0.3 | 13.5 ± 0.3 | | 3 |  | −14.5 ± 0.4 | | 12.0 ± 0.3 | 5 |
| *Oratosquilla aratoria* (Japanese mantis shrimp) | −15.2 ± 0.7 | 11.5 ± 0.7 | | 3 |  | −14.6 ± 0.6 | | 15.0 ± 0.7 | 5 |
| *Pagurus* sp. (Hermit crab) | −15.7 | 10.4 | | 1 |  | −16.4 | | 14.7 |  |
| *Palaemon gravieri* (Chinese ditch prawn) | −14.3 ± 0.4 | 11.1 ± 0.4 | | 2 |  |  | |  |  |
| *Parapenaeopsis tenella* (Smooth shell shrimp) | −15.1 ± 0.3 | 13.6 ± 0.2 | | 2 |  | −14.6 ± 0.8 | | 12.9 ± 0.7 | 4 |
| *Philyra pisum* (Pea pebble crab) | −14.8 ± 0.1 | 10.8 ± 0.3 | | 2 |  |  | |  |  |
| *Philyra* sp. (Pebble crab) |  |  | |  |  | −13.1 | | 11.9 | 1 |
| *Portunus trituberculatus* (Swimming crab) | −15.6 ± 1.8 | 12.7 ± 0.4 | | 2 |  | −14.9 ± 0.3 | | 12.4 ± 0.3 | 2 |
| *Trachysalambria curvirostris* (Cocktail shrimp) | −15.1 ± 0.9 | 12.9 ± 0.4 | | 2 |  |  | |  |  |
| Mixed zooplankton | −20.5 ± 0.2 | 10.1 ± 0.9 | | 2 |  |  | |  |  |
| Echinodermata |  |  | |  |  |  | |  |  |
| Stelloridea |  |  | |  |  |  | |  |  |
| *Asterias amurensis* (North Pacific seastar) |  |  | |  |  | −19.0 | | 13.8 | 1 |
| Echinodea |  |  | |  |  |  | |  |  |
| *Hemicentrotus* sp. (Sea urchin) |  |  | |  |  | −20.0 | | 12.2 | 1 |
| Holothuroidea |  |  | |  |  |  | |  |  |
| *Protankyra bidentata* (Bidentate sea cucumber) | −14.4 ± 0.4 | 12.6 ± 0.4 | | 3 |  | −13.7 | | 14.1 | 1 |
| Fish |  |  | |  |  |  | |  |  |
| *Acanthogobius flavimanus* (Genuine goby) | −14.0 | 16.7 | | 1 |  |  | |  |  |
| *Acanthopagrus schlegeli* (Black sea bream) | −14.6 ± 0.3 | 10.2 ± 0.2 | | 2 |  |  | |  |  |
| *Amblychaeturichthys hexanema* (Pinkgray goby) | −14.7 ± 0.1 | 12.7 ± 0.8 | | 3 |  | −14.5 ± 0.7 | | 13.8 ± 0.3 | 5 |
| *Apogon lineatus* (Vertical striped cardinalfish) |  |  | |  |  | −15.2 ± 0.1 | | 13.5 ± 0.4 | 4 |
| *Chelidonichthys spinosus* (Bluefin sea robin) | −16.5 | 11.2 | | 1 |  | −14.9 ± 0.2 | | 11.8 ± 0.1 | 2 |
| *Cociella crocodila* (Spotted flathead) | −15.3 | 15.1 | | 1 |  |  | |  |  |
| *Cryptocentrus filifer* (Gafftopsail goby) | −14.7 ± 0.4 | 14.4 ± 0.4 | | 2 |  |  | |  |  |
| *Cynoglossus abbreviatus* (Short nose tongue sole) | −13.8 ± 0.2 | 14.5 ± 0.5 | | 2 |  |  | |  |  |
| *Cynoglossus joyneri* (Red tongue sole) | −15.4 | 13.3 | | 1 |  |  | |  |  |
| *Cynoglossus robustus* (Robust tonguefish) | −14.3 | 13.6 | | 1 |  |  | |  |  |
| *Cynoglossus* sp. (Sole) |  |  | |  |  | −13.9 ± 0.2 | | 13.8 ± 0.7 | 5 |
| *Decapterus maruadsi* (White -tipped mackerel scad) | −16.8 | 11.3 | | 1 |  |  | |  |  |
| *Engraulis japonicus* (Anchovy) | −16.9 | 11.4 | | 1 |  |  | |  |  |
| *Inimicus japonicus* (Devil stinger) |  |  | |  |  | −13.8 ± 0.4 | | 15.5 ± 0.3 | 2 |
| *Konosirus punctatus* (Dotted gizzard shad) |  |  | |  |  | −14.2 | | 14.6 | 1 |
| *Larimichthys polyactis* (Yellow croaker) | −14.7 ± 0.1 | 15.2 ± 0.2 | | 2 |  | −14.7 ± 0.5 | | 15.2 ± 0.7 | 5 |
| *Leiognathus nuchalis* (Spot nape ponyfish) | −15.6 ± 0.4 | 11.8 ± 0.8 | | 3 |  | −15.9 ± 0.9 | | 14.2 ± 0.4 | 3 |
| *Mugil cephalus* (Gray mullet) | −14.9 | 13.9 | | 1 |  |  | |  |  |
| *Odontamblyopus lacepedii* (Green eel goby) |  |  | |  |  | −13.6 ± 0.1 | | 15.6 ± 0.6 | 3 |
| *Pampus argenteus* (Silver pomfret) | −15.1 ± 0.2 | 15.4 ± 0.3 | | 3 |  | −15.6 ± 0.2 | | 14.5 ± 0.0 | 3 |
| *Paralichthys olivaceus* (Olive flounder) | −17.4 | 12.6 | | 1 |  |  | |  |  |
| *Pennahia argentata* (White croaker) | −15.0 ± 0.4 | 13.9 ± 0.2 | | 2 |  |  | |  |  |
| *Platycephalus indicus* (Bartail flathead) | −14.5 ± 0.4 | 14.6 ± 1.0 | | 3 |  | −15.2 ± 0.4 | | 14.1 ± 0.7 | 5 |
| *Pleuronichthys cornutus* (Fine-spotted flounder) |  |  | |  |  | −14.0 ± 0.3 | | 13.8 ± 0.1 | 3 |
| *Pseudoblennius cottoides* (Sunrise sculpin) | −12.9 | 16.4 | | 1 |  |  | |  |  |
| *Repomucenus sagitta* (Arrow dragonet) | −15.2 | 13.5 | | 1 |  |  | |  |  |
| *Sardinella zunasi* (Big-eyed herring) | −14.4 | 14.4 | | 1 |  |  | |  |  |
| *Scomber japonicus* (Conger pike) | −15.2 ± 0.9 | 11.4 ± 0.7 | | 3 |  | −14.4 ± 0.7 | | 14.7 ± 0.5 | 4 |
| *Sebastes inermis* (Dark-banded rockfish) | −14.3 | 14.9 | | 1 |  |  | |  |  |
| *Sillago sihama* (Sand smelt) | −14.9 ± 0.7 | 11.0 ± 0.7 | | 3 |  |  | |  |  |
| *Takifugu* sp. (Puffer) | −15.4 | 13.0 | | 1 |  |  | |  |  |
| *Thryssa adelae* (Short-horned anchovy) | −15.1 | 15.4 | | 1 |  | −16.3 ± 0.9 | | 14.2 ± 0.0 | 2 |
| *Thryssa kammalensis* (Anchovy) | −15.8 ± 0.3 | 15.2 ± 0.3 | | 3 |  | −16.5 ± 0.1 | | 14.0 ± 0.5 | 4 |
| *Upeneus japonicus* (Yellow-fin goatfish) | −14.6 ± 0.2 | 14.5 ± 0.3 | | 3 |  |  | |  |  |
