# Supplementary material for: Linking Intertidal and Subtidal Food Webs: Consumer-Mediated Transport of Intertidal Benthic Microalgal Carbon
Source: PLoS One. 2015 Oct 8;10(10):e0139802. doi: 10.1371/journal.pone.0139802 (PMC4598165; doi:10.1371/journal.pone.0139802)
Supplement: S2 Appendix — All samples were pooled by each taxon (zooplankton) or species (other consumers). (DOC) [file pone.0139802.s002.doc]

| Species name | Spring | |  | Fall | |
| --- | --- | --- | --- | --- | --- |
|  | δ^13^C | δ^15^N |  | δ^13^C | δ^15^N |
| Zooplankton |  |  |  |  |  |
| Amphipods |  |  |  | −20.3 | 7.5 |
| Copepods | −21.2 | 7.4 |  | −21.5 | 6.4 |
| Euphausiids | −21.3 | 6.2 |  | −19.6 | 7.0 |
| Chaetognaths (Arrow worm) | −20.7 | 8.3 |  | −20.3 | 7.7 |
|  |  |  |  |  |  |
| Arthropoda |  |  |  |  |  |
| Crustacea |  |  |  |  |  |
| *Alpheus japonicas* (Japanese snapping shrimp) |  |  |  | −16.7 | 10.1 |
| *Amphiura* sp. |  |  |  | −17.5 | 9.0 |
| *Carcinoplax longimana* (Long armed crab) |  |  |  | −16.1 | 9.5 |
| *Charybdis bimaculata* (Two-spot swimming crab) |  |  |  | −17.5 | 9.3 |
| *Charybdis(Charybdis) sagamiensis* (Sagami swimming crab) | −16.1 | 10.9 |  |  |  |
| *Dardanus arrosor* (Gaforre heremite) | −16.3 | 10.6 |  |  |  |
| *Ibacus ciliates* (Slipper lobster) | −16.1 | 10.5 |  |  |  |
| *Ibacus novemdentatus* (Smooth fan lobster) | −15.9 | 11.2 |  |  |  |
| *ZoLeptomithrax edwardsii* (Edsards’ spider crab) | −18.0 | 8.9 |  |  |  |
| *Liocarcinus corrugatus* (Wrinkled swimming crab) | −17.0 | 9.9 |  |  |  |
| *Metapenaeopsis provocatoria longirostris* (Penaeid shrimp) | −16.9 | 8.6 |  |  |  |
| *Munida japonica* (Japanese squat lobster) | −17.5 | 8.8 |  |  |  |
| Mollusca |  |  |  |  |  |
| Cephalopoda |  |  |  |  |  |
| *Loligo beka* (Beka squid) |  |  |  | −16.0 | 13.4 |
| *Loligo edulis* (Swordtip squid) | −17.0 | 11.2 |  |  |  |
| *Octopus ocellatus* (Webfoot octopus) | −17.3 | 8.1 |  |  |  |
| *Octopus vulgaris* (Common octopus) | −16.2 | 11.0 |  | −17.2 | 10.4 |
| *Sepia esculenta* (Golden cuttlefish) | −17.0 | 11.8 |  | −16.2 | 11.3 |
| *Sepiella japonica* (Japanese spineless cuttlefish) | −17.1 | 10.3 |  |  |  |
| Sepiidae unid. | −17.6 | 9.0 |  |  |  |
| *Todarodes pacificus* (Japanese flying squid) | −18.7 | 8.8 |  | −17.3 | 11.0 |
| Fish |  |  |  |  |  |
| *Argentina kagoshimae* (Smoothback angelshark) | −16.6 | 12.0 |  |  |  |
| *Aulopus japonicas* (Japanese aulopus) | −17.5 | 10.2 |  | −16.6 | 11.9 |
| *Bembras japonica* (Red flathead) | −16.8 | 11.5 |  |  |  |
| *Champsodon snyderi* (Gaper) | −16.8 | 8.0 |  |  |  |
| *Conger myriaster* (Conger eel) |  |  |  | −17.6 | 11.9 |
| *Coryphaena hippurus* (Common dolphinfish) | −17.7 | 12.6 |  |  |  |
| *Dentex tumifrons* (Yellowback seabream) | −16.5 | 13.2 |  | −16.6 | 12.0 |
| *Dipturus kwangtungensis* (Kwangtung skate) | −16.1 | 11.0 |  |  |  |
| *Doederleinia berycoides* (Blackthroat seaperch) |  |  |  | −18.4 | 11.2 |
| *Echelus uropterus* (Finned worm eel) | −16.8 | 12.3 |  |  |  |
| *Engraulis japonicas* (Anchovy) | −18.0 | 10.8 |  |  |  |
| *Eopsetta grigorjewi* (Shotted halibut) | −16.6 | 10.8 |  |  |  |
| *Glossanodon semifasciatus* (Deep sea smelt) |  |  |  | −17.4 | 10.0 |
| *Glyptocephalus stelleri* (Blackfin flounder) |  |  |  | −16.2 | 10.8 |
| *Gnathagnus elongates* (Bluespotted stargazer) |  |  |  | −16.8 | 12.6 |
| *Gnathagnus nystrominystromi* (Conger ell) | −17.2 | 9.7 |  |  |  |
| *Halieutaea stellate* (Starry handfish) | −16.4 | 11.6 |  |  |  |
| *Helicolenus hilgendorfi* (Rosefish) |  |  |  | −17.7 | 11.2 |
| *Hoplobrotula armata* (Armored brotula) |  |  |  | −18.0 | 10.6 |
| *Kaiwarinus equula* (Whitefin trevally) | −16.5 | 12.5 |  | −16.8 | 11.8 |
| *Lepidotrigla abyssalis* (Abyssal searobin) | −17.2 | 10.6 |  |  |  |
| *Lepidotrigla guentheri* (Gurnard) |  |  |  | −17.3 | 10.9 |
| *Lepidotrigla hime* (Gurnard) | −16.7 | 11.7 |  |  |  |
| *Lophiomus setigerus* (Blackmouth angler) | −16.0 | 13.3 |  |  |  |
| *Lophius litulon* (Yellow goosefish) | −17.1 | 11.2 |  | −16.7 | 12.0 |
| *Macroramphosus scolopax* (Snipefish) | −17.0 | 10.4 |  |  |  |
| *Muraenesox cinereus* (Daggertooth pike conger) |  |  |  | −16.6 | 12.9 |
| *Niphon spinosus* (Sawedged) |  |  |  | −17.5 | 11.4 |
| *Okamejei acutispina* (Sharpspine skate) | −16.9 | 10.5 |  |  |  |
| *Okamejei meerdervoortii* (Bigeye skate) | −17.2 | 10.5 |  |  |  |
| *Oplegnathus fasciatus* (Striped beakfish) | −16.2 | 12.7 |  |  |  |
| *Pagrus major* (Red seabream) | −15.8 | 13.6 |  |  |  |
| *Parapristipoma trilineatum* (Chicken grunt) |  |  |  | −18.6 | 9.6 |
| *Pleuronichthys cornutus* (Fine-spotted flounder) | −16.2 | 11.6 |  |  |  |
| *Psenopsis anomala* (Butterfish) |  |  |  | −17.4 | 11.8 |
| *Sardinops melanostictus* (Spotlined sardine) |  |  |  | −17.0 | 10.8 |
| *Saurida microlepis* (Shortfin lizardfish) | −16.5 | 12.6 |  |  |  |
| *Saurida wanieso* (Wanieso lizardfish) | −16.8 | 14.2 |  |  |  |
| *Scomber japonicus* (Conger pike) |  |  |  | −18.1 | 11.5 |
| *Scomberomorus niphonius* (Japanese Spanish mackerel) |  |  |  | −17.3 | 13.8 |
| *Scorpaena neglecta* (Izu scorpionfish, sting fish) | −16.1 | 11.9 |  | −17.8 | 13.2 |
| *Sphyraena japonica* (Japanese barracuda) |  |  |  | −15.5 | 12.6 |
| *Synagrops japonicas* (Blackmouth spiltfin) | −17.2 | 11.6 |  |  |  |
| *Synodus macrops* (Triplecross lizardfish) | −16.9 | 12.3 |  | −16.9 | 11.5 |
| *Thamnaconus modestus* (Black scraper) | −16.2 | 12.0 |  | −16.3 | 10.6 |
| *Thunnus thynnus* (Atlantic bluefin tuna) |  |  |  | −17.3 | 12.5 |
| *Trachurus japonicas* (Japanese jack mackerel) | −17.5 | 11.8 |  | −17.2 | 11.9 |
| *Trichiurus lepturus* (Largehead hairtail) | −17.6 | 11.5 |  | −17.2 | 11.0 |
| *Upeneus japonicas* (Yellow-fin goatfish) | −16.8 | 12.4 |  |  |  |
| *Uranoscopus japonicas* (Japanese stargazer) |  |  |  | −16.3 | 12.5 |
| *Zebrias zebra* (Zebra sole) |  |  |  | −17.4 | 11.7 |
| *Zenopsis nebulosa* (Mirror dory) | −16.1 | 12.6 |  | −16.7 | 10.6 |
| *Zeus faber* (John dory) | −17.1 | 11.6 |  | −16.1 | 12.3 |
